# Supplementary material for: Functional validation of somatic variability in TP53 and KRAS for prediction of platinum sensitivity and prognosis in epithelial ovarian carcinoma patients
Source: Cancer Biol Ther. 2025 Aug 10;26(1):2543105. doi: 10.1080/15384047.2025.2543105 (PMC12341051; doi:10.1080/15384047.2025.2543105)
Supplement: KCBT_S_2025_0041R1_Online_Resouces.docx [file KCBT_A_2543105_SM8423.docx]

**Online Resources**

*Molecular Diagnosis & Therapy*

**Functional validation of somatic variability in *TP53* and *KRAS* for prediction of platinum sensitivity and prognosis in epithelial ovarian carcinoma patients**

Mohammad Al Obeed Allah^1^, Esraa Alli^1^, Ivona Krus^2^, Petr Holý^1,2^, Vojtěch Haničinec^1^, Filip Ambrozkiewicz^1^, Lukáš Rob^3^, Martin Hruda^3^, Marcela Mrhalová^4^, Kateřina Kopečková^5^, Alena Bartáková^6^, Jiří Bouda^6^, Spálenková Alžběta^1,2^, Pavel Souček^1,2^, and Radka Václavíková^1,2^*

^1^Biomedical Center, Faculty of Medicine in Pilsen, Charles University, Pilsen, Czech Republic;

^2^Toxicogenomics Unit, National Institute of Public Health, Prague, Czech Republic;

^3^Department of Gynecology and Obstetrics, Third Faculty of Medicine, Charles University and University Hospital Královské Vinohrady, Prague, Czech Republic;

^4^Department of Pathology and Molecular Medicine, Second Faculty of Medicine, Charles University and Motol University Hospital, Prague, Czech Republic;

^5^Department of Oncology and Molecular Medicine, Second Faculty of Medicine, Charles University and Motol University Hospital, Prague, Czech Republic;

^6^Department of Gynecology and Obstetrics, Faculty of Medicine and University Hospital in Pilsen, Charles University, Pilsen, Czech Republic.

*****Correspondence: Radka Václavíková, Biomedical Center, Faculty of Medicine in Pilsen, Charles University, Pilsen 306 05, Czech Republic; email: radka.vaclavikova@lfp.cuni.cz, phone: +420 267 082 711; ORCID: 0000-0002-0451-4725

**List of Online Resources**

**Online Resource 1** Associations between stage (A), residuum after surgery (B), and chemosensitivity status (C) and overall survival of EOC patients

**Online Resource 2** Representative chromatograms of *KRAS* mutations assessed in EOC patients by direct Sanger sequencing

A – codon 12 in exon 2, B – codon 61 in exon 3

**Online Resource 3** Representative chromatograms of *TP53* mutations assessed in EOC patients by direct Sanger sequencing

A – p.Pro75fs, B – p.Arg175His, C – p.His179Gln, D – p.His214Arg, E – p.Tyr220Cys, F – p.Glu198Ter, G – p.Arg213Ter, H – p.Asp259Tyr, I – p.Arg273His, J – p.Arg282Trp, K – p.Arg248His/Trp.

**Online Resource 4** Detailed mutational data

**Online Resource 5** Associations between EOC subtype and disease stage

**Online Resource 6** Association between subtype and platinum-free (A) and overall (B) survival of EOC patients

**Online Resource 7** Schematic picture of exons and list of primers used for *KRAS* and *TP53* mutation analysis in EOC patients by direct Sanger sequencing

**Online Resource 8** Immunoblots with KRAS and p53 protein analysis

**Online Resource 1** Associations between stage (A), residuum after surgery (B), and chemosensitivity status (C) and overall survival of EOC patients


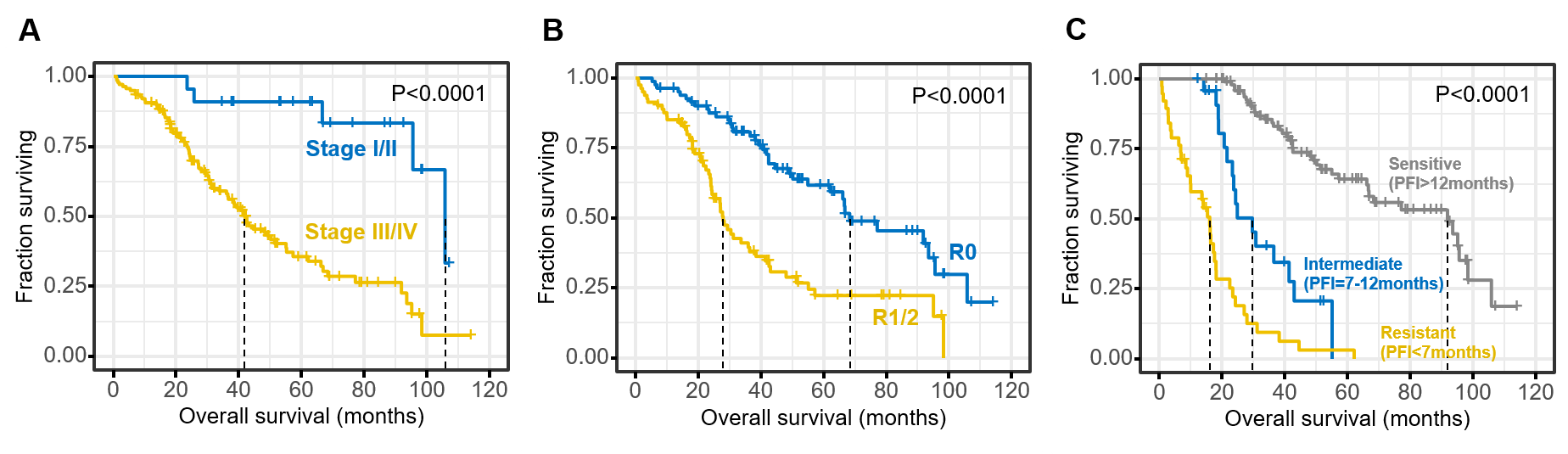


**Online Resource 2** Representative chromatograms of *KRAS* mutations assessed in EOC patients by direct Sanger sequencing

A – codon 12 in exon 2, B – codon 61 in exon 3


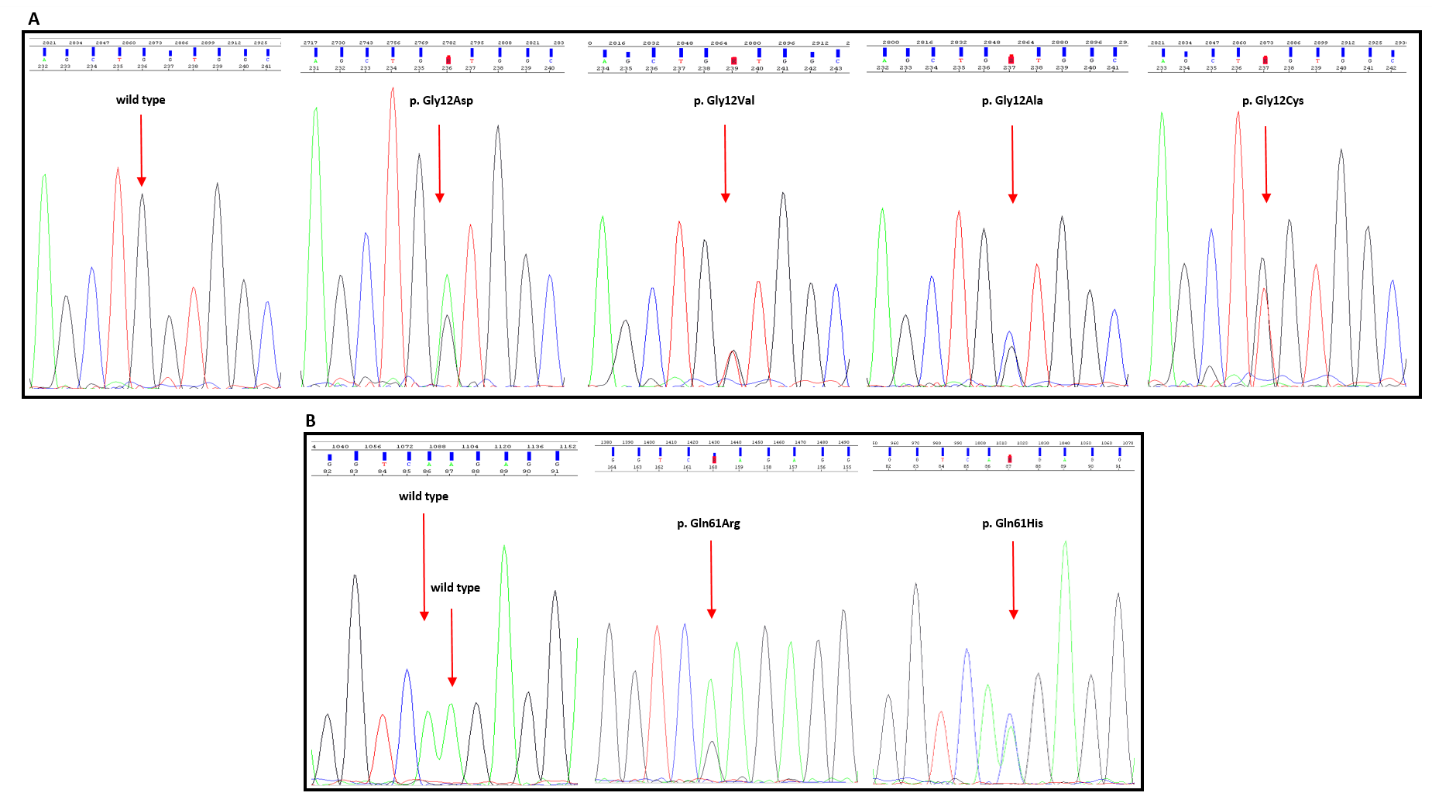


**Online Resource 3** Representative chromatograms of *TP53* mutations assessed in EOC patients by direct Sanger sequencing

A – p.Pro75fs, B – p.Arg175His, C – p.His179Gln, D – p.His214Arg, E – p.Tyr220Cys, F – p.Glu198Ter, G – p.Arg213Ter, H – p.Asp259Tyr, I – p.Arg273His, J – p.Arg282Trp, K – p.Arg248His/Trp.


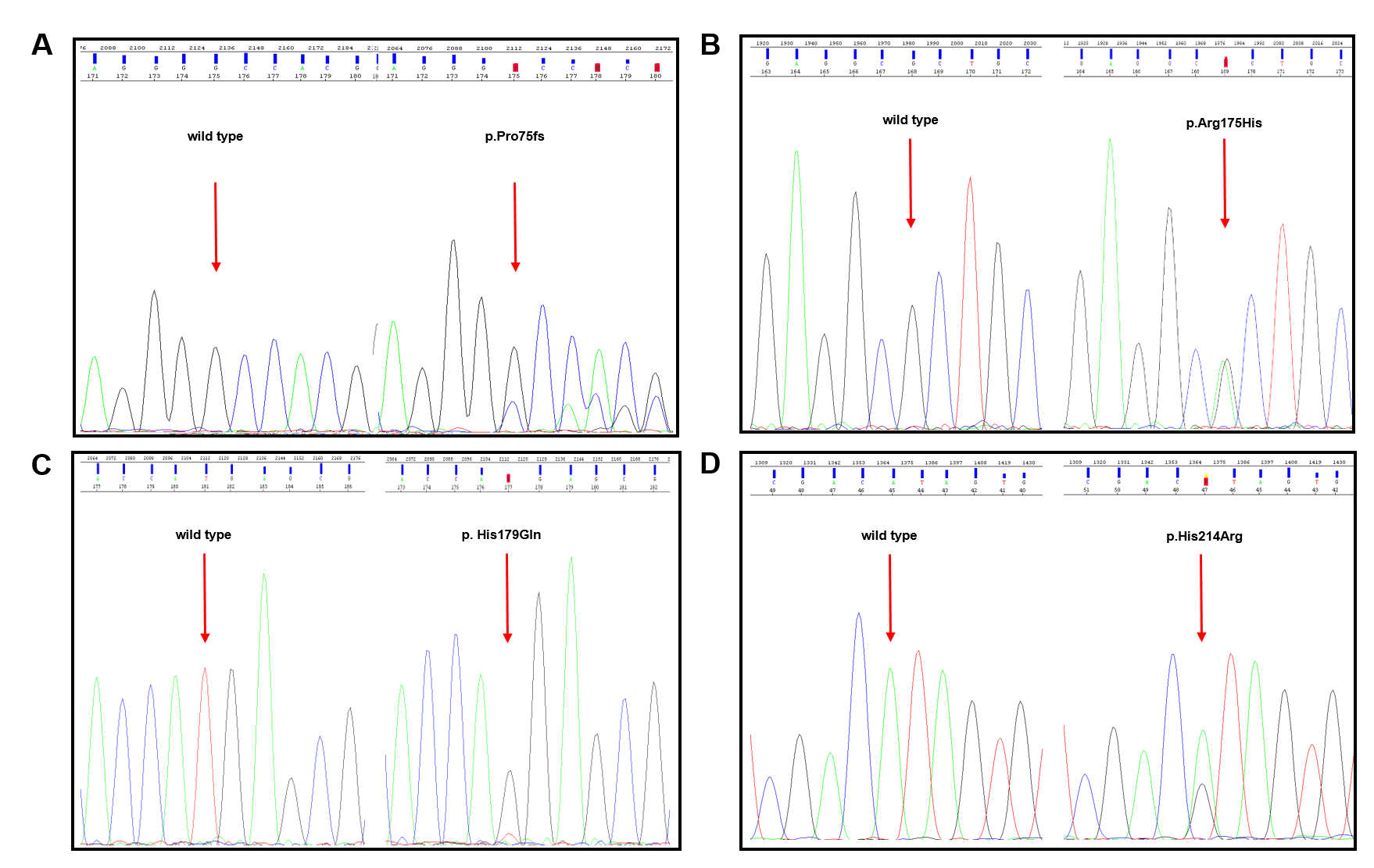


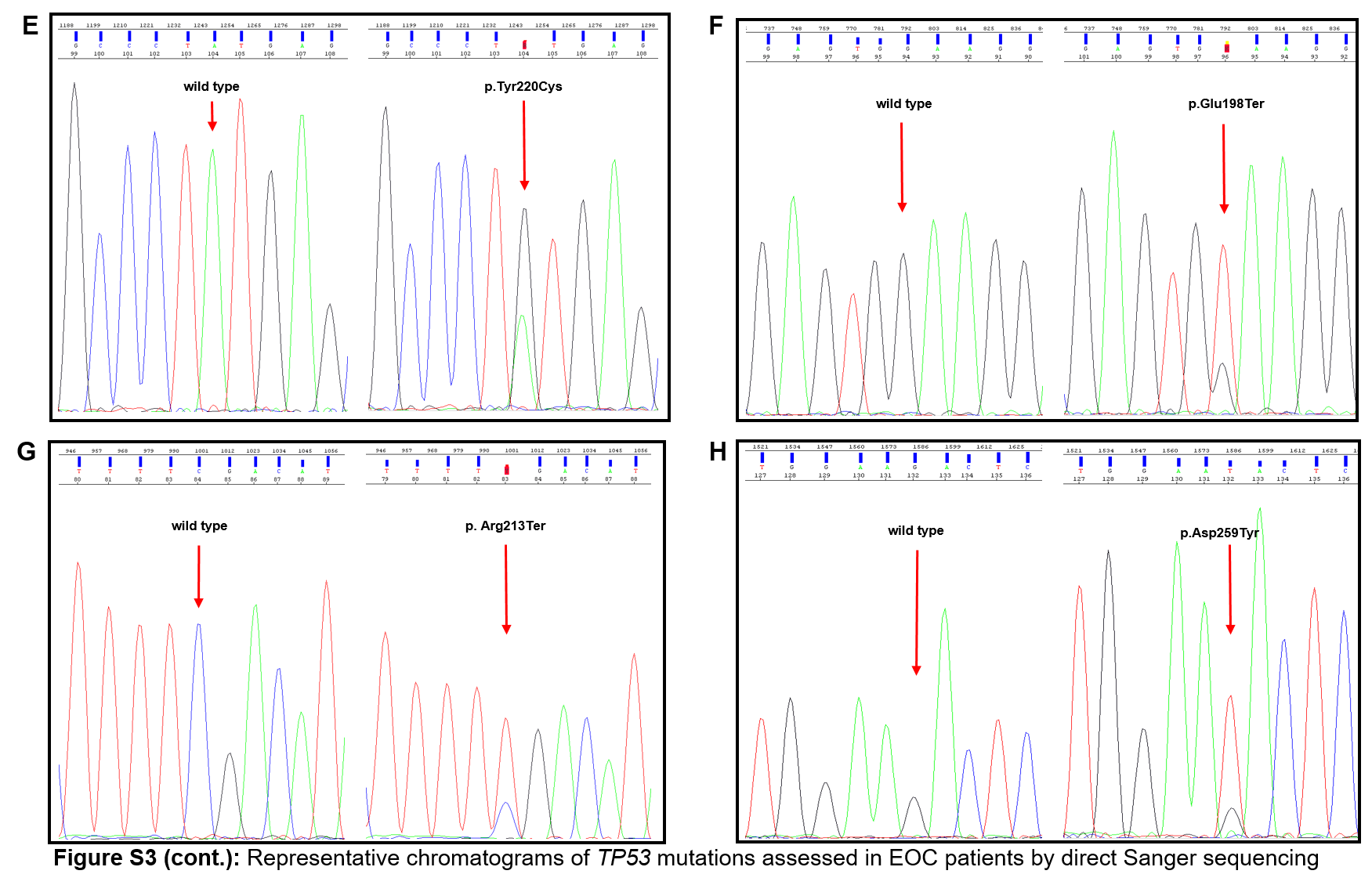


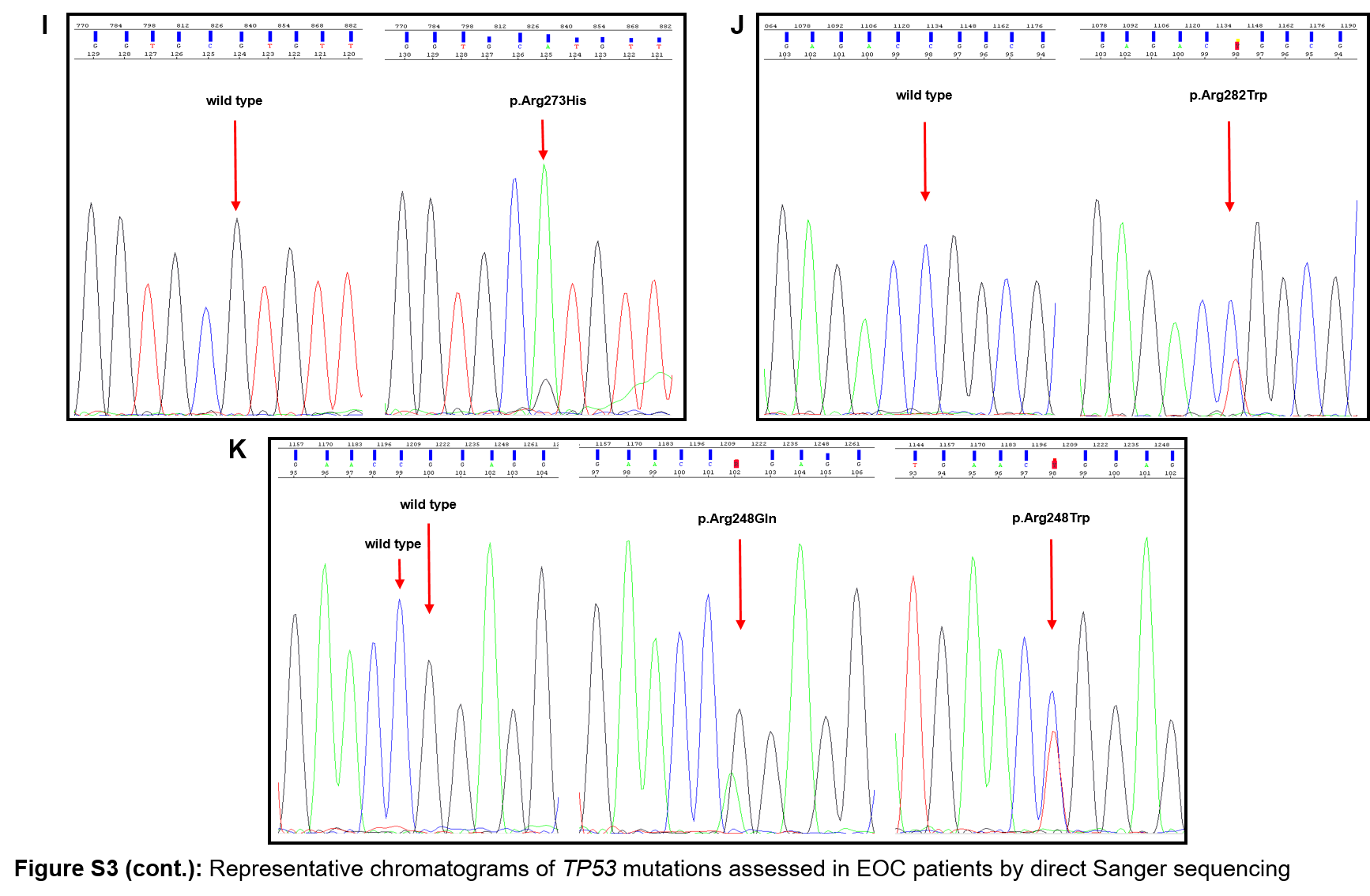


**Online Resource 4** Detailed mutational data

| Sample ID | *KRASmut* | *TP53mut* | *GoF/LoF* | *DNA binding loops https://tp53.isb-cgc.org/* | *DNE and/or LOF https://tp53.isb-cgc.org/* | *Transactivation https://tp53.isb-cgc.org/* |
| --- | --- | --- | --- | --- | --- | --- |
| PL2 | wild type | p.Arg282Trp | LoF | affected | DNE_LOF | non-functional |
| PL3 | wild type | p.Pro316SerfsTer21 | LoF | not affected | NA | NA |
| PL11 | wild type | p.Cys176Phe | LoF | affected | DNE_LOF | partially functional |
| PL16 | wild type | c.993+2T>G, c.993+1G>T, Splice donor variant | NA | NA | NA | NA |
| PL17 | wild type | wild type | wild type | wild type | wild type | wild type |
| PL18 | wild type | p.Arg273His | LoF | affected | DNE_LOF | non-functional |
| PL20 | wild type | wild type | wild type | wild type | wild type | wild type |
| PL23 | wild type | p.Glu198* | LoF | affected | notDNE_notLOF | NA |
| PL24 | wild type | p.Arg273His | LoF | affected | DNE_LOF | non-functional |
| PL25 | wild type | p.Leu264_Asn268del | NA | affected | NA | NA |
| PL27 | wild type | p.Arg273His | LoF | affected | DNE_LOF | non-functional |
| PL29 | p.Gln61His | wild type | wild type | wild type | wild type | wild type |
| PL31 | p.Gly12Val | p.Arg282Trp | LoF | affected | DNE_LOF | non-functional |
| PL32 | wild type | c.993+12T>C=intronic - no effect | NA | NA | NA | NA |
| PL33 | wild type | c.993+12T>C=intronic - no effect | NA | NA | NA | NA |
| PL34 | wild type | p.Arg175His | LoF | affected | DNE_LOF | non-functional |
| PL37 | wild type | p.X224_splice | NA | NA | NA | NA |
| PL41 | wild type | p.Arg175His | LoF | affected | DNE_LOF | non-functional |
| PL45 | p.Gly12Asp | wild type | wild type | wild type | wild type | wild type |
| PL47 | wild type | p.His214Arg | LoF | affected | DNE_LOF | non-functional |
| PL51 | wild type | wild type | wild type | wild type | wild type | wild type |
| PL55 | wild type | p.Arg283AlafsX112 | LoF | affected | NA | NA |
| PL56 | wild type | wild type | wild type | wild type | wild type | wild type |
| PL57 | p.Gly12Val | wild type | wild type | wild type | wild type | wild type |
| PL62 | wild type | p.Arg175His | LoF | affected | DNE_LOF | non-functional |
| PL66 | wild type | wild type | wild type | wild type | wild type | wild type |
| PL70 | wild type | p.Arg248Gln | GoF | affected | DNE_LOF | non-functional |
| PL74 | wild type | wild type | wild type | wild type | wild type | wild type |
| PL83 | p.Gly12Val | wild type | wild type | wild type | wild type | wild type |
| PL84 | wild type | wild type | wild type | wild type | wild type | wild type |
| PL86 | wild type | p.Asp228fs | LoF | affected | NA | NA |
| PL87 | wild type | p.Val157Phe | LoF | affected | unclassified | non-functional |
| PL88 | wild type | p.Cys135Tyr | LoF | affected | DNE_LOF | non-functional |
| R4 | wild type | wild type | wild type | wild type | wild type | wild type |
| R6 | wild type | wild type | wild type | wild type | wild type | wild type |
| R7 | wild type | p.Arg306* | LoF | not affected | notDNE_LOF | NA |
| R11 | NA | p.Phe338LeufsTer8 | LoF | not affected | NA | NA |
| R15 | wild type | wild type | wild type | wild type | wild type | wild type |
| R17 | p.Gln61Arg | p.Arg248Gln | GoF | affected | DNE_LOF | non-functional |
| R21 | p.Gly12Asp | wild type | wild type | wild type | wild type | wild type |
| R22 | wild type | wild type | wild type | wild type | wild type | wild type |
| R27 | wild type | wild type | wild type | wild type | wild type | wild type |
| R33 | wild type | c.673-36G>C=intronic - no effect | NA | NA | NA | NA |
| R35 | wild type | wild type | wild type | wild type | wild type | wild type |
| R43 | wild type | p.Arg282Gly | LoF | affected | DNE_LOF | non-functional |
| R44 | wild type | c.672+1G>A | NA | NA | NA | NA |
| R45 | wild type | p.Pro75fs | LoF | not affected | NA | NA |
| R47 | wild type | wild type | wild type | wild type | wild type | wild type |
| R61 | wild type | p.Arg175His | LoF | affected | DNE_LOF | non-functional |
| R66 | wild type | wild type | wild type | wild type | wild type | wild type |
| R71 | p.Gly12Val | wild type | wild type | wild type | wild type | wild type |
| R76 | wild type | p.Gly279Glu | LoF | affected | notDNE_LOF | non-functional |
| R77 | p.Gly12Asp | wild type | wild type | wild type | wild type | wild type |
| R79 | wild type | p.Thr155fs | LoF | affected | NA | NA |
| R88 | wild type | wild type | wild type | wild type | wild type | wild type |
| R89 | wild type | wild type | wild type | wild type | wild type | wild type |
| R90 | wild type | p.Gly279Glu | LoF | affected | notDNE_LOF | non-functional |
| R92 | wild type | p.Val274Phe | LoF | affected | DNE_LOF | non-functional |
| R93 | wild type | wild type | wild type | wild type | wild type | wild type |
| R96 | wild type | wild type | wild type | wild type | wild type | wild type |
| R97 | wild type | c.e7-1G>T canonical splice site | NA | NA | NA | NA |
| R98 | wild type | p.Gly245Asp | LoF | affected | unclassified | non-functional |
| R99 | wild type | p.Arg273His | LoF | affected | DNE_LOF | non-functional |
| R102 | wild type | p.Arg175His | LoF | affected | DNE_LOF | non-functional |
| R103 | wild type | wild type | wild type | wild type | wild type | wild type |
| R105 | wild type | wild type | wild type | wild type | wild type | wild type |
| R107 | p.Gly12Val | p.Cys135Trp | LoF | affected | DNE_LOF | partially functional |
| R109 | wild type | p.Val173Leu | LoF | affected | unclassified | non-functional |
| R110 | wild type | p.Arg248Gln | GoF | affected | DNE_LOF | non-functional |
| R111 | p.Gly12Asp | wild type | wild type | wild type | wild type | wild type |
| R112 | wild type | p.Arg175His | LoF | affected | DNE_LOF | non-functional |
| R114 | wild type | p.Cys238Phe | LoF | affected | DNE_LOF | non-functional |
| R115 | p.Gly12Asp | p.Arg213* | LoF | affected | notDNE_LOF | NA |
| R117 | wild type | p.His214Arg | LoF | affected | DNE_LOF | non-functional |
| R118 | wild type | p.Lys132Arg | LoF | affected | DNE_LOF | non-functional |
| R119 | wild type | wild type | wild type | wild type | wild type | wild type |
| R121 | wild type | p.Arg213Thr | LoF | affected | DNE_LOF | NA |
| R122 | wild type | p.Tyr163Cys | LoF | affected | DNE_LOF | non-functional |
| R123 | wild type | p.Leu265del | NA | affected | NA | NA |
| R127 | wild type | p.Arg248Gln | GoF | affected | DNE_LOF | non-functional |
| R128 | wild type | p.Val272Leu | LoF | affected | DNE_LOF | non-functional |
| R131 | p.Gln61His | wild type | wild type | wild type | wild type | wild type |
| R133 | wild type | wild type | wild type | wild type | wild type | wild type |
| R136 | wild type | wild type | wild type | wild type | wild type | wild type |
| R137 | wild type | c.993+2T>G, Splice donor variant | NA | NA | NA | NA |
| R139 | wild type | p.Thr211Ile | LoF | affected | DNE_LOF | partially functional |
| R141 | wild type | p.Arg248Trp | LoF | affected | DNE_LOF | non-functional |
| R143 | wild type | p.Arg213AspfsTer34 | LoF | affected | NA | NA |
| R144 | wild type | p.Arg213* | LoF | affected | notDNE_LOF | NA |
| R145 | wild type | p.Arg248Trp | LoF | affected | DNE_LOF | non-functional |
| R148 | wild type | p.Arg282Trp | LoF | affected | DNE_LOF | non-functional |
| R149 | wild type | 7675238delT | NA | NA | NA | NA |
| R150 | wild type | p.Glu286Lys | LoF | affected | DNE_LOF | non-functional |
| R152 | wild type | p.Arg248Trp | LoF | affected | DNE_LOF | non-functional |
| R153 | wild type | p.Arg248Gln | GoF | affected | DNE_LOF | non-functional |
| R154 | wild type | p.Glu198* | LoF | affected | notDNE_notLOF | NA |
| R155 | wild type | p.Cys275Phe | LoF | affected | DNE_LOF | non-functional |
| R159 | wild type | p.Asp207LysfsTer49 | LoF | affected | NA | NA |
| R160 | wild type | p.Met246Val | LoF | affected | DNE_LOF | non-functional |
| R162 | wild type | p.Leu308ArgfsTer37 | LoF | not affected | NA | NA |
| R163 | wild type | p.Thr256HisfsTer89 | LoF | affected | NA | NA |
| R164 | wild type | p.Arg282del=in frame del | NA | affected | NA | NA |
| R167 | wild type | p.Glu224Glu=silent | NA | NA | NA | NA |
| R168 | wild type | wild type | wild type | wild type | wild type | wild type |
| R212 | wild type | wild type | wild type | wild type | wild type | wild type |
| R213 | wild type | p.Tyr205Asp | LoF | affected | DNE_LOF | non-functional |
| R214 | wild type | p.Ala161Thr | GoF | affected | DNE_LOF | partially functional |
| R215 | wild type | p.Tyr220Cys | LoF | affected | DNE_LOF | non-functional |
| R216 | p.Gly12Cys | wild type | wild type | wild type | wild type | wild type |
| R218 | wild type | wild type | wild type | wild type | wild type | wild type |
| R229 | wild type | wild type | wild type | wild type | wild type | wild type |
| R230 | wild type | p.Val274LeufsX121 | LoF | affected | NA | NA |
| R233 | wild type | p.His179Gln | LoF | affected | DNE_LOF | non-functional |
| R234 | wild type | p.Ser269LeufsX125 | LoF | affected | NA | NA |
| R235 | wild type | wild type | wild type | wild type | wild type | wild type |
| R237 | wild type | wild type | wild type | wild type | wild type | wild type |
| R238 | wild type | wild type | wild type | wild type | wild type | wild type |
| R240 | wild type | p.Gly245Ala | LoF | affected | DNE_LOF | non-functional |
| R241 | wild type | p.Gly244Asp | LoF | affected | DNE_LOF | non-functional |
| R243 | wild type | p.Arg273His | LoF | affected | DNE_LOF | non-functional |
| R245 | wild type | wild type | wild type | wild type | wild type | wild type |
| R247 | wild type | p.Tyr220Cys | LoF | affected | DNE_LOF | non-functional |
| R249 | wild type | p.His179Asp | LoF | affected | DNE_LOF | partially functional |
| R250 | wild type | p.Asn239GlnfsX155 | LoF | affected | NA | NA |
| R252 | wild type | p.Tyr220Cys | LoF | affected | DNE_LOF | non-functional |
| R254 | wild type | p.Tyr220Cys, p.Arg273His | LoF | affected | DNE_LOF | non-functional |
| R256 | wild type | wild type | wild type | wild type | wild type | wild type |
| R257 | wild type | p.Arg175His | LoF | affected | DNE_LOF | non-functional |
| R258 | wild type | p.Val173Met | LoF | affected | DNE_LOF | non-functional |
| R260 | wild type | p.His179Arg | LoF | affected | DNE_LOF | non-functional |
| R261 | wild type | wild type | wild type | wild type | wild type | wild type |
| R262 | wild type | p.Gln192* | LoF | affected | notDNE_notLOF | NA |
| R263 | wild type | wild type | wild type | wild type | wild type | wild type |
| R264 | wild type | p.His179Gln | LoF | affected | DNE_LOF | partially functional |
| R265 | wild type | p.Tyr220Cys | LoF | affected | DNE_LOF | non-functional |
| R266 | wild type | p.Phe134LeufsX260 | LoF | affected | NA | NA |
| R267 | wild type | wild type | wild type | wild type | wild type | wild type |
| R270 | wild type | wild type | wild type | wild type | wild type | wild type |
| R271 | wild type | wild type | wild type | wild type | wild type | wild type |
| R272 | wild type | wild type | wild type | wild type | wild type | wild type |
| R273 | wild type | p.Tyr220Cys | LoF | affected | DNE_LOF | non-functional |
| R274 | wild type | p.Ser127Phe | LoF | affected | DNE_LOF | non-functional |
| R276 | wild type | p.Arg248Trp | LoF | affected | DNE_LOF | non-functional |
| R277 | wild type | p.Pro142fs | LoF | affected | NA | NA |
| R278 | wild type | p.Tyr234Cys | LoF | affected | DNE_LOF | non-functional |
| R279 | p.Gly12Ala | wild type | wild type | wild type | wild type | wild type |
| R282 | wild type | p.Tyr236Cys | LoF | affected | DNE_LOF | non-functional |
| R283 | wild type | wild type | wild type | wild type | wild type | wild type |
| R284 | wild type | p.Cys275Phe | LoF | affected | DNE_LOF | non-functional |
| R285 | wild type | wild type | wild type | wild type | wild type | wild type |
| R286 | wild type | wild type | wild type | wild type | wild type | wild type |
| R287 | wild type | wild type | wild type | wild type | wild type | wild type |
| R288 | wild type | p.Tyr220Cys | LoF | affected | DNE_LOF | non-functional |
| R290 | wild type | wild type | wild type | wild type | wild type | wild type |
| R291 | wild type | wild type | wild type | wild type | wild type | wild type |
| R292 | wild type | p.Arg249Trp | GoF | affected | DNE_LOF | non-functional |
| R293 | wild type | p.Thr256del=in frame del | NA | affected | NA | NA |
| R295 | wild type | p.Glu198* | LoF | affected | notDNE_notLOF | NA |
| R296 | wild type | p.Arg249Trp | GoF | affected | DNE_LOF | non-functional |
| R298 | wild type | p.His214Arg | LoF | affected | DNE_LOF | non-functional |
| R300 | wild type | wild type | wild type | wild type | wild type | wild type |
| R302 | wild type | p.Arg175His | LoF | affected | DNE_LOF | non-functional |
| R309 | wild type | wild type | wild type | wild type | wild type | wild type |
| R310 | wild type | wild type | wild type | wild type | wild type | wild type |
| R311 | wild type | p.Arg273His | LoF | affected | DNE_LOF | non-functional |
| R312 | wild type | p.Leu206Met | NA | affected | notDNE_notLOF | functional |
| R321 | wild type | wild type | wild type | wild type | wild type | wild type |
| R327 | wild type | p.Asp259Tyr | LoF | affected | DNE_LOF | non-functional |
| R329 | wild type | wild type | wild type | wild type | wild type | wild type |
| R330 | wild type | p.Arg282Trp | LoF | affected | DNE_LOF | non-functional |
| R331 | wild type | p.Arg342* | LoF | not affected | notDNE_LOF | NA |
| R334 | wild type | wild type | wild type | wild type | wild type | wild type |
| R335 | wild type | wild type | wild type | wild type | wild type | wild type |
| R345 | wild type | wild type | wild type | wild type | wild type | wild type |
| R346 | wild type | wild type | wild type | wild type | wild type | wild type |
| R347 | wild type | wild type | wild type | wild type | wild type | wild type |
| R348 | wild type | wild type | wild type | wild type | wild type | wild type |

Abbreviations: fs=frameshift

NA=not available

**Online Resource 5** Associations between EOC subtype and disease stage

| **Characteristics** | **HGSC subtype*** | **nonHGSC subtypes*** | **p-value** |
| --- | --- | --- | --- |
| Stage I/II | 13 | 13 | <0.001 |
| Stage III/IV | 132 | 8 |  |

Footnotes:

*Numbers of patients; for some patients clinical data were not available.

**Online Resource 6** Association between subtype and platinum-free (A) and overall (B) survival of EOC patients


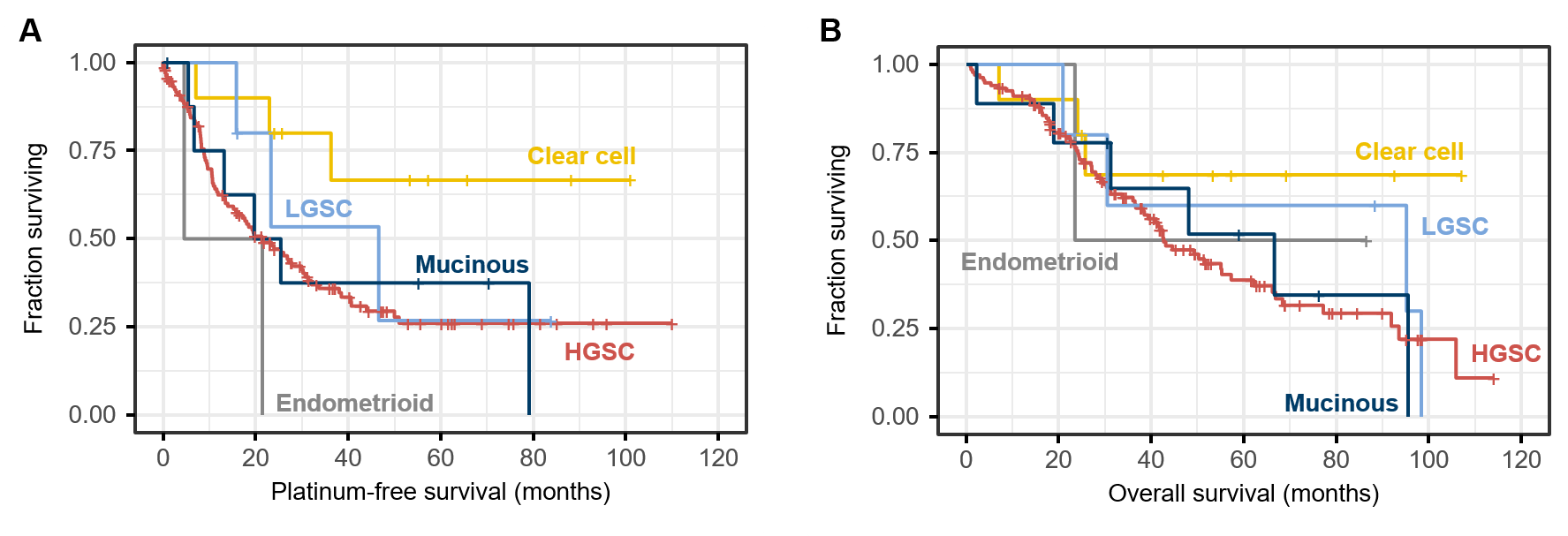


**Online Resource 7** Schematic picture of exons and list of primers used for *KRAS* and *TP53* mutation analysis in EOC patients by direct Sanger sequencing

| **Gene/Exon** |  | **Sequencing primers (5’->3’)** | | |  |
| --- | --- | --- | --- | --- | --- |
|  | Forward | | Location (GRCh38.p14) | Reverse | Location (GRCh38.p14) |
| *TP53* |  | |  |  |  |
| 4 | CCCAGGGTTGGAAGTGTCTC | | 7676671-7676652 | TGAGACTTCAATGCCTGGCC | 7675965-7675946 |
| 5 | CACTTGTGCCCTGACTTTCA | | 7675282-7675263 | AGAGACGACAGGGCTGGTT | 7675034-7675016 |
| 6 | CAGGCCTCTGATTCCTCACT | | 7675003-7674984 | CTCTGGGAGGAGGGGTTAAG | 7674838-7674819 |
| 7 | CCACAGGTCTCCCCAAGG | | 7674345-7674328 | CTGCACACTGGCCTGCTG | 7674160-7674143 |
| 8 | GCCTCTTGCTTCTCTTTTCC | | 7673869-7673850 | GGAGACCAAGGGTGCAGTTA | 7673672-7673653 |
| 9 | AGCACTAAGCGAGGTAAGCA | | 7673713-7673694 | CCAGGAGCCATTGTCTTTGA | 7673394-7673413 |
| 10 | TGCATGTTGCTTTTGTACCGT | | 7670823-7670803 | GGCTGGGACCCAATGAGAT | 7670483-7670501 |
| *KRAS* |  | |  |  |  |
| 2 | AAGCGTCGATGGAGGAGTTT | | 25245616-25245597 | GAATGGTCCTGCACCAGTAAT | 25245230-25245250 |
| 3 | AGGTGCACTGTAATAATCCAGACT | | 25227454-25227431 | TGCATGGCATTAGCAAAGACTC | 25227152-25227173 |

**
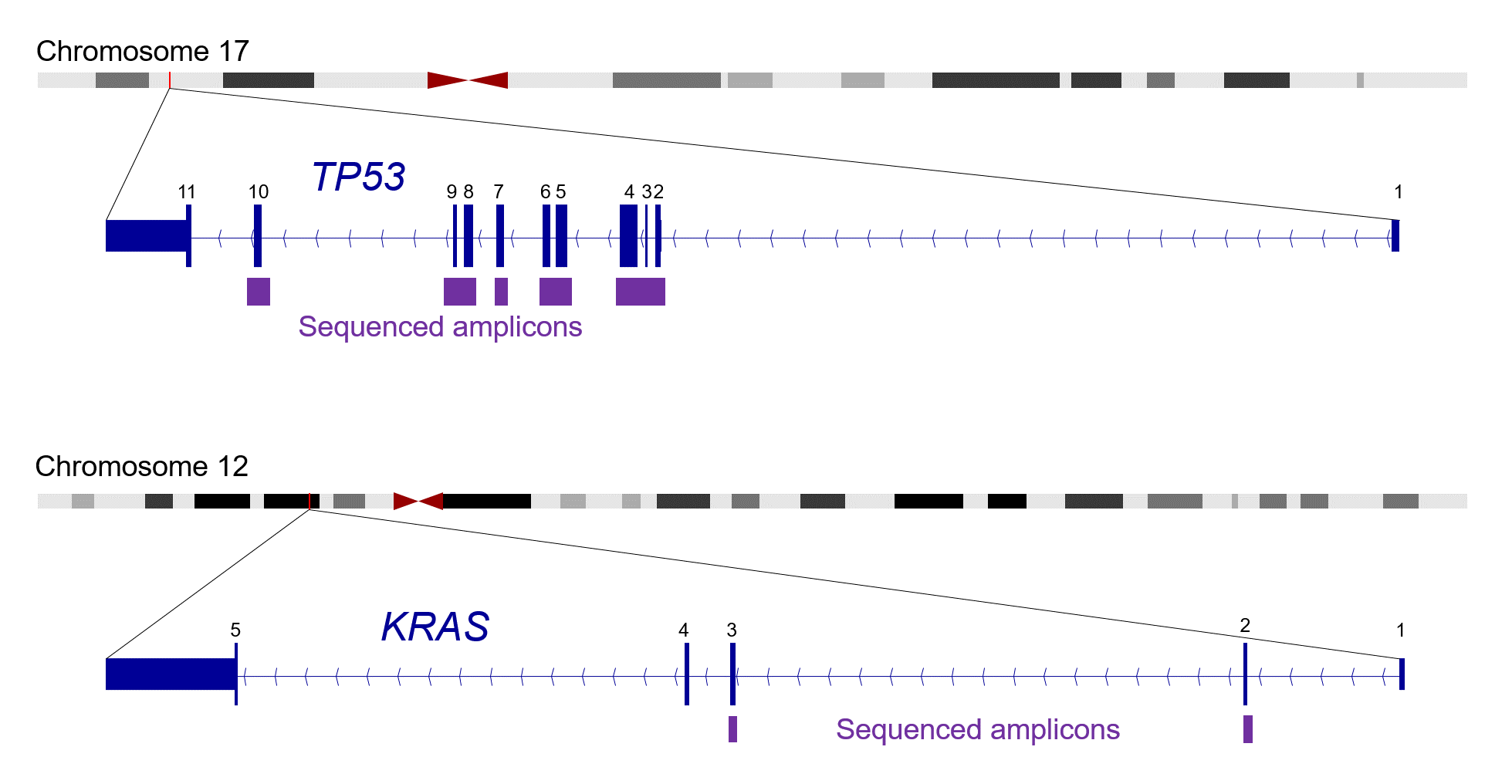
**

**Online Resource 8** Immunoblots with KRAS and p53 protein analysis

**
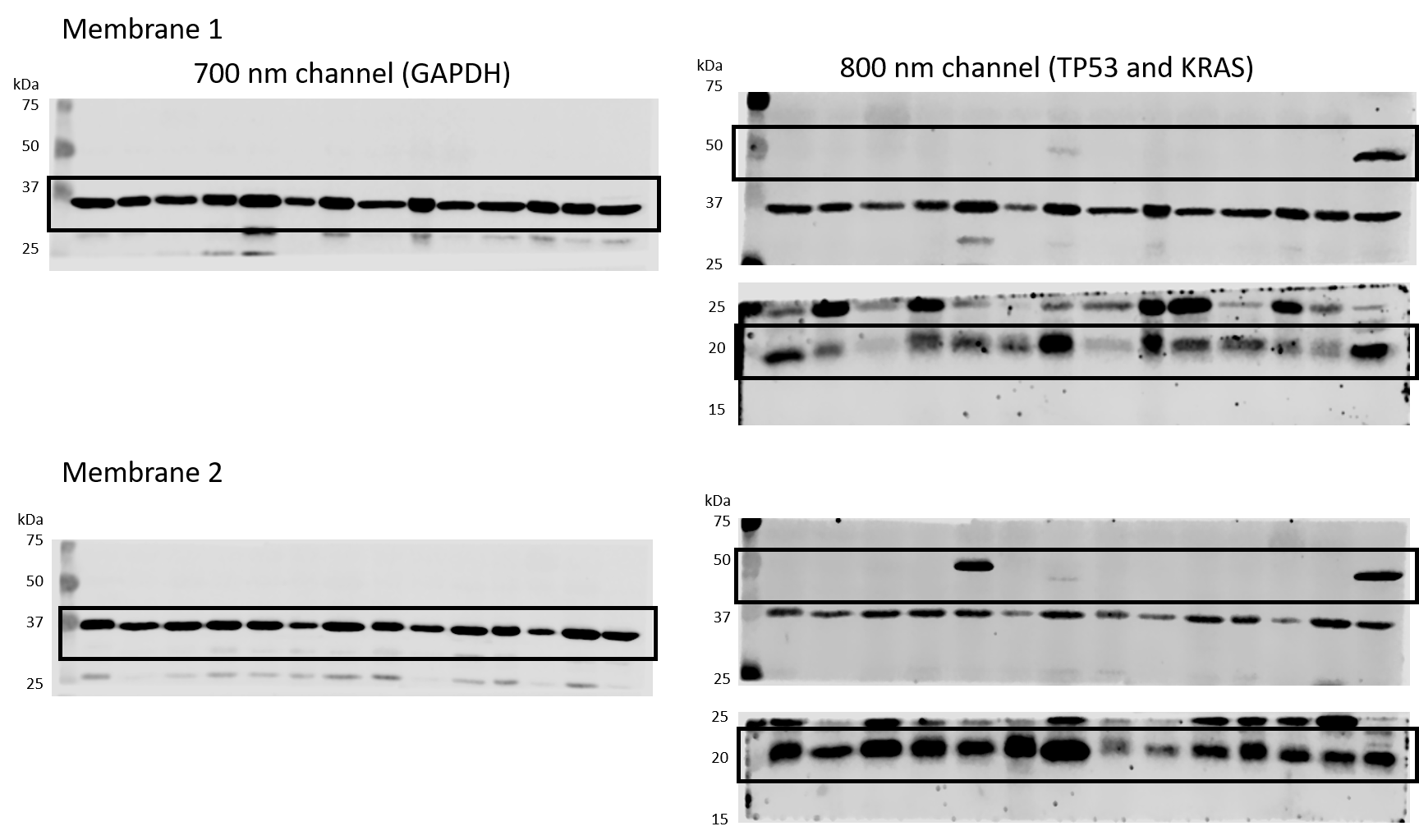
**

**
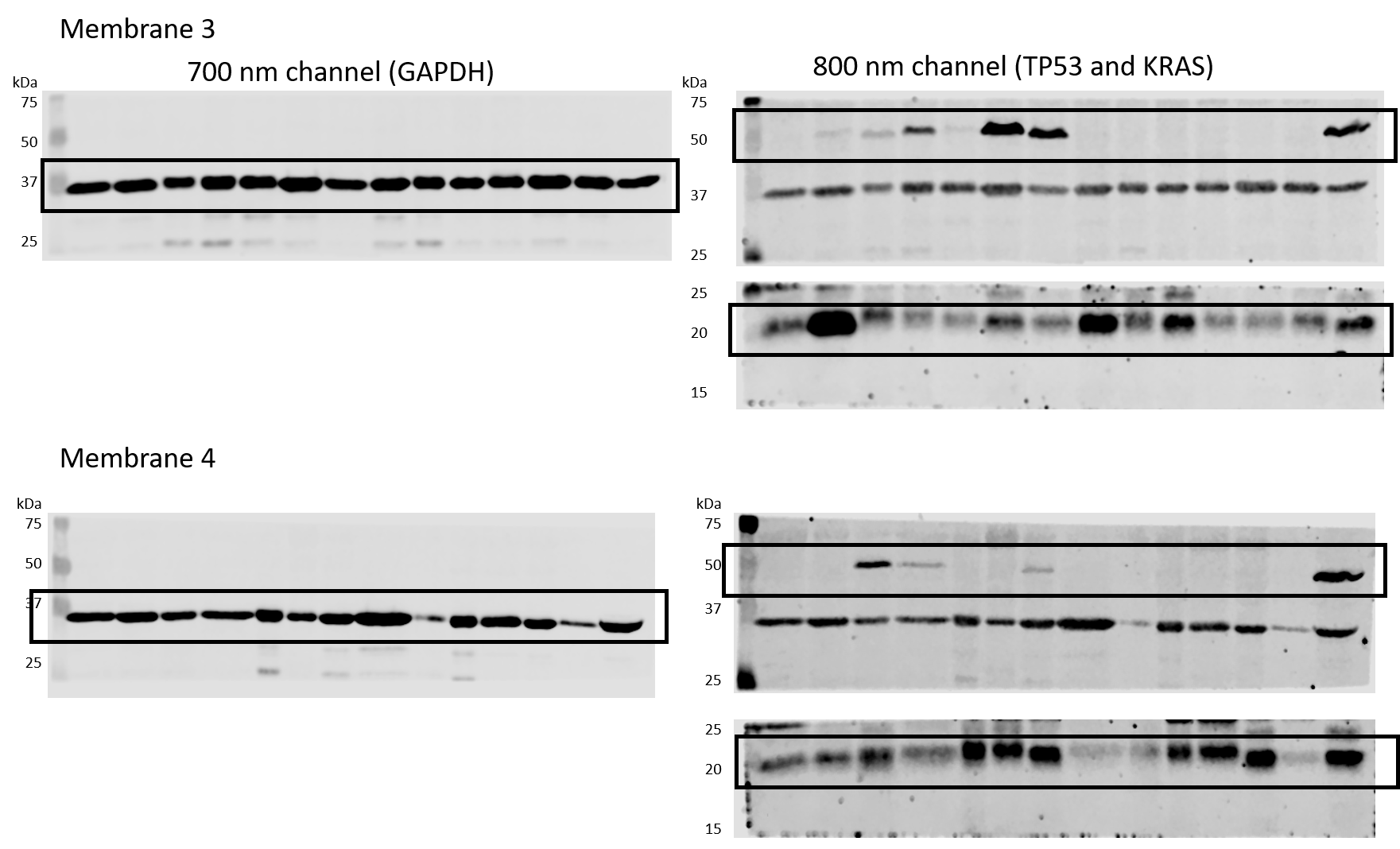
**
