# Supplementary material for: Functional validation of somatic variability in TP53 and KRAS for prediction of platinum sensitivity and prognosis in epithelial ovarian carcinoma patients
Source: Cancer Biol Ther. 2025 Aug 10;26(1):2543105. doi: 10.1080/15384047.2025.2543105 (PMC12341051; doi:10.1080/15384047.2025.2543105)
Supplement: Manuscript clean version R1.docx [file KCBT_A_2543105_SM8422.docx]

**Functional validation of somatic variability in *TP53* and *KRAS* for prediction of platinum sensitivity and prognosis in epithelial ovarian carcinoma patients**

*Al Obeed Allah Mohammad^1^, Ali Esraa^1^, Krus Ivona ^2^, Holý Petr^1,2^, Haničinec Vojtěch^1^, Ambrozkiewicz Filip^1^, Rob Lukáš^3^, Hruda Martin^3^, Mrhalová Marcela^4^, Kopečková Kateřina^5^, Bartáková Alena^6^, Bouda Jiří^6^, Spálenková Alžběta^1,2^, Souček Pavel^1,2*^, and Václavíková Radka^1,2^*

^1^Biomedical Center, Faculty of Medicine in Pilsen, Charles University, Pilsen, Czech Republic;

^2^Toxicogenomics Unit, National Institute of Public Health, Prague, Czech Republic;

^3^Department of Gynecology and Obstetrics, Third Faculty of Medicine, Charles University and University Hospital Královské Vinohrady, Prague, Czech Republic;

^4^Department of Pathology and Molecular Medicine, Second Faculty of Medicine, Charles University and Motol University Hospital, Prague, Czech Republic;

^5^Department of Oncology and Molecular Medicine, Second Faculty of Medicine, Charles University and Motol University Hospital, Prague, Czech Republic;

^6^Department of Gynecology and Obstetrics, Faculty of Medicine and University Hospital in Pilsen, Charles University, Pilsen, Czech Republic.

*****Pavel Souček, National Institute of Public Health, Srobarova 49/48, Prague, 10000, Czech Republic, email: pavel.soucek@szu.cz, phone: +420 267 082 711; ORCID: 0000-0002-4294-6799

**Abstract**

*Background:* Concerning the dismal prognosis of chemoresistant patients with epithelial ovarian carcinoma (EOC), we aimed to follow up the findings of a previous whole-exome sequencing study using an orthogonal Sanger sequencing on the same patients and a separate set of 127 EOC patients (N=177, all fresh frozen tumor samples).

*Methods:* We focused on *TP53* as a frequently mutated gene relevant for chemosensitivity, included *KRAS* as an additional therapeutically relevant target, complemented the study with transcript levels of both genes, and compared results with clinical parameters.

*Results:* All variants in *TP53* and *KRAS* detected by exome sequencing were confirmed. *KRAS* mutated patients had significantly more frequent FIGO stages I or II (p=0.002) and other than high-grade serous tumor subtypes (nonHGSCs) (p<0.001), which was connected with lower KRAS transcript levels (p=0.004). Patients with nonHGSC subtypes had less frequent *TP53* mutations (p=0.002). Carriers of *TP53* variants disrupting the DNA binding loop had significantly longer platinum-free intervals than the rest (p=0.037). Tumors bearing nonsense, frameshift, or splice site *TP53* variants had a significantly lower TP53 transcript level, while those with missense variants had significantly higher levels than wild types (p<0.001). The normalized intratumoral TP53 and KRAS transcript levels were correlated, and patients with co-mutated genes had poorer overall survival than others (p=0.015). Protein levels of both genes significantly correlated with their respective transcripts (p=0.028 and p=0.001, respectively).

*Conclusion*: Our study points to *KRAS* as a target for future therapy of nonHGSCs and reveals the prognostic value of *TP53* variants in the DNA binding loop.

**Keywords:** epithelial ovarian carcinoma; platinum sensitivity; TP53; KRAS; variant; transcript expression

**Running head**: Somatic TP53 and KRAS variability in ovarian cancer

**Introduction**

Epithelial ovarian cancer (EOC), recognized as the eighth leading cause of cancer-related death among women, stands out as one of the most lethal gynecological malignancies [1]. Early detection of EOC is a challenge, because, in most cases, it is asymptomatic in the early stages (I or II based on The International Federation of Gynecology and Obstetrics, FIGO, guidelines). Typically, 75% of EOC cases occur at the advanced stage (III or IV), where the 5-year survival rate is approximately 20–45%, compared to 40–70% for stages I or II [2,3]. The standard treatment for advanced EOC has been primary debulking surgery followed by chemotherapy (platinum derivatives with paclitaxel) for the majority of cases [4]. However, most patients experience a relapse within the first five years after the initial diagnosis, with only 20-25% achieving cure [5].

Morphologically, EOCs are classified into four major subtypes: serous, endometrioid, clear cell, and mucinous [6]. Additionally, they can be divided into two primary types: type I, including endometrioid, mucinous, clear cell, and low-grade serous ovarian carcinomas (LGSCs), and type II, constituting 70% of the total and encompassing high-grade serous ovarian carcinomas (HGSCs), carcinosarcomas, and undifferentiated carcinomas. These classifications are integral to defining the aggressiveness of the cancer and its response to different chemotherapies [3]. A significant majority, exceeding 80% of identified EOC cases, fall under the histological classification of HGSC, characterized by an aggressive phenotype that correlates with elevated mortality rate [7], which is attributed not only to diagnosis at the advanced stage but also to chemoresistance, where approximately 50% of the cases diagnosed at advanced stage relapse within the first five years [3].

The introduction of Poly(ADP-ribose) polymerase (PARP) inhibitors like olaparib and antiangiogenic agents, such as bevacizumab or pazopanib, has led to a significant improvement in the prognosis of the patients [8,9]. PARP inhibitors (PARPis) are primarily used for maintenance therapy for platinum-sensitive advanced EOCs [10]. Moreover, patients with *BRCA1*/*BRCA2* mutations demonstrate enhanced sensitivity to treatment with PARPi [11].

Next Generation Sequencing (NGS) enables the detection of genetic variability and its linkage to multidrug resistance. Based on genomic profiling, two major EOC types have been defined. EOC of type I is characterized by mutations in the MAPK pathway (*KRAS, BRAF, PTEN,* and *CTNNB1*, etc.) and type II mutations in *TP53, BRCA1, BRCA2, KIT*, and *EGFR* [12]. Moreover, DNA damage response and related alterations in DNA repair pathways play a crucial role in cancer development, including EOC. Germline mutations in DNA repair genes can predict hereditary forms of cancer, particularly *BRCA1/2* mutations in breast and ovarian cancers [13]. Pathogenic somatic mutations in genes from the homologous recombination DNA repair pathway, such as *BRCA1/2*, *ATM*, *RAD51C*, and *RAD51D*, were implicated in chemosensitivity and prognosis of EOC patients [14,15]. HGSC typically shows very high frequency of somatic *TP53* mutations (~90 %) and genomic heterogeneity [16].

A previous whole exome sequencing study [14] confirmed *TP53* as the most frequently mutated gene in HGSC and EOC in general and suggested its relevance for chemosensitivity. Moreover, the followed sample set enriched with clear cell, mucinous, endometrioid, and low grade serous carcinomas quite high frequency of mutations in *KRAS* (overall 12%) [14], suggesting its relevance for therapeutic decisions. The present follow up study uses direct Sanger sequencing of the same and an extended sample sets to demonstrate the robustness of *TP53* and *KRAS* mutation detection, provide validation of study results, and substantiate their further use in clinical management of patients. We also complement somatic mutation screening with an assessment of both genes’ transcript levels in tumor RNA and protein levels in selected cases. We compare the results with sensitivity to EOC therapy and patient survival for evaluation of the prognostic value of these biomarkers. Our study adds another dimension to exome or genome sequencing-based EOC projects published before [14, 17-19].

**Results**

*Patients’ characteristics*

The main characteristics of all patients (N=177) are in **Table 1**. The median age of patients at the time of diagnosis was 62 years (range 24–89). Most patients presented with FIGO stage III (82%), grade G3 (85%), and HGSC subtype (84%). About one-third of patients (32%) underwent preoperative chemotherapy, and half of patients (50%) had disease residuum left after surgical tumor debulking. The vast majority of patients (96%) received platinum-based chemotherapy regimens in an adjuvant setting, two received taxane monotherapy, four did not receive any adjuvant treatment due to poor performance status, and for six patients the information about therapy was not available. The median PFI and OS were 25 and 48 months, respectively. Patients with FIGO stage III or IV, residuum after surgery (R1 or R2), or with PFI <12 months had significantly poorer OS than the rest of the patients (p<0.001 for all) (**Online Resource 1A-C**).

*Somatic genetic variability*

All six *KRAS* variants found previously by exome sequencing (n=50) were also detected by Sanger sequencing in the confirmation part of the study (n=50). In the extended validation part (n=125, two samples not assessed due to the lack of DNA), variants in a further nine samples were observed (**Table 2**). All variants were missense single nucleotide substitutions in exon 2 (n=12) or 3 (n=3). Representative chromatograms are in **Online Resource 2A, B**.

As for *TP53*, the confirmation set showed exactly the same variants compared to exome sequencing, i.e., 39 mutated and 10 wild-type patients. In the validation set (n=127), an additional 66 mutated samples were identified (**Table 2**). Representative chromatograms are presented in **Online Resource 3A-K**.

Functional classifications enabled the distribution of TP53 variants to several categories: i/ missense (n=77), out of which 32 were single private mutations and the rest mutational hotspots detected in two or more patients, ii/ two hotspot nonsense variants present in three patients each, and iii/ private frameshifts or nonsense variants (n=17). The last category was splice site variants with pathogenic features, which were all private (n=5). The TP53 database of NCI and The Clinical Knowledgebase (CKB) enabled more detailed stratification of variants into loss-of-function (n=87) versus gain-of-function (n=8) variants. Most of the somatic variants were classified as having the following properties: dominant-negative effect or loss-of-function (n=66), non-functional transactivation (n=64), and affecting DNA binding loop (n=94) by these databases (**Table 2, Online Resource 4**).

Four patients carried mutations in both *TP53* and *KRAS* (co-mutations). One patient with the HGSC subtype had the combination of *TP53*-Arg282Trp with *KRAS*-Gln61His, chemoresistant status, and OS of 16 months. The second patient had a clear cell subtype, *TP53*-Arg248Gln with *KRAS*-Gln61Arg, chemoresistant status, and extremely short OS of 7 months. The third patient with the mucinous subtype had *TP53*-Arg213Ter with *KRAS*-Gly12Asp mutation combination, chemoresistant status, and OS of 19 months. The last patient had *TP53*-Cys135Trp with KRAS- Gly12Val, HGSC subtype, chemosensitive status, and OS of 43 months. Thus, carriage of *TP53-KRAS* co-mutations could be associated with chemoresistance and poor patient prognosis in most cases observed.

All subsequent clinical genomic analyses were performed using the combined confirmation and validation cohorts (N=177).

*Intratumoral KRAS and TP53 transcript and protein levels*

To provide additional functional evidence, we analyzed by qPCR the TP53 and KRAS transcript levels in all available tumor samples together with genetic information. Five samples could not be determined due to low RNA quantity or quality and no tissue left. No extreme outliers were observed.

The carriage or type of *KRAS* mutations did not significantly associate with the KRAS or TP53 transcript levels (p>0.05). On the other hand, a significantly lower TP53 transcript level in tumors bearing nonsense, frameshift, or splice site types of variants compared to wild-type TP53 was observed (p<0.001, **Fig. 1A**). In contrast, tumors with missense *TP53* variants had significantly higher transcript levels than wild-type ones (p<0.001, **Fig. 1A**). Higher TP53 transcript level was found in tumors with *TP53* variants classified as gain-of-function compared to loss-of-function (p=0.027, **Fig. 1B**).

Carriage of co-mutated *TP53-KRAS* did not affect transcript expression (p=0.096 for TP53 and p=0.306 for KRAS).

Interestingly, the normalized intratumoral TP53 and KRAS transcript levels were mutually significantly correlated (ρ=0.384, p<0.001, **Fig. 1C**). Additionally, protein level of KRAS (**Fig 1D**), analyzed by immunoblotting, significantly correlated with that of transcript (n=52, ρ=0.305 and p=0.028) and for p53 protein (**Fig 1D**) with transcript such correlation was even stronger (n=52, ρ=0.431 and p=0.001). KRAS and p53 protein levels did not correlate at all (p=0.804).

*3.4 Associations of somatic genetic variability and transcript levels with clinical data of patients*

Afterward, we performed statistical analysis of associations between transcript levels, mutational status, spectra, and functional classifications of both genes and clinical data of patients.

Patients with FIGO stage I or II had significantly more frequently mutated *KRAS* compared to stage III or IV patients (p=0.002, **Table 3**). On the other hand, patients with the HGSC tumor subtype had significantly less frequently mutated *KRAS* and *TP53* (p<0.001 and p=0.002, respectively; **Table 3**), and they had significantly higher KRAS transcript levels (p=0.004, **Fig. 1E**) compared to those with other EOC subtypes. On the other hand, KRAS transcript level did not associate with its mutation status (p=0.181, **Fig. 1F**). *KRAS* mutation status, spectra, or transcript level were not significantly associated with the rest of the clinical parameters (age, grade of tumor, surgical radicality, chemosensitivity status, or PFI and OS, all p>0.05), and this was true for the association between transcript level and stage as well.

As for *TP53,* its transcript level, mutation status, spectra, or functional classifications were not significantly associated with any of the clinical parameters (age, stage, grade of tumor, subtype, surgical radicality, chemosensitivity status, or OS, all p>0.05). However, the PFI of patients with *TP53* mutations non-disrupting the DNA binding loop (n=6) was significantly poorer than that of patients with mutations located in the domain (p=0.037, **Fig. 2A)**. Patients with co-mutated *TP53*-*KRAS* had significantly worse OS than wild-type patients or those with a single gene mutation (p=0.015, **Fig. 2B**), but the PFI was not significantly affected (p=0.065, **Fig. 2C**).

We further performed patient stratification into HGSC and nonHGSC subgroups, given the importance of the EOC subtype in previous analyses. Patients with nonHGSC subtypes had significantly more often less advanced stages I/II than HGSCs (p<0.001, **Online Resource 5**) and thus less aggressive disease. However, only the PFI of patients with clear cell subtype (n=10) was any better than that of HGSC (n=134), while for mucinous (n=9) or LGSC (n=5), it was not, and endometrioid patients had worst PFI (n=2) (**Online Resource 6A**). The above differences between subtypes had no prognostic consequences (**Online Resource 6B**).

No significant associations with clinical data were identified for KRAS or TP53 transcript levels, mutations, or their functional classifications in the HGSC subgroup (n=143). However, patients with nonHGSC subtypes (n=28) bearing any *TP53* mutations had non-significantly poorer PFI than patients with the wild-type (p=0.062). No association was found for OS or other clinical data, including chemosensitivity status.

*Validation using external datasets*

Finally, we attempted to validate our findings using the largest and most up-to-date publicly available EOC dataset within the GENIE project (n=2210).

The *TP53* and *KRAS* mutation analysis confirmed the overrepresentation of *KRAS* mutations in nonHGSC compared to HGSC cases. In our dataset, 32% of nonHGSC patients harbored *KRAS* mutations, while only 1.4% of HGSC cases had such alterations. Size of the GENIE dataset allowed the analysis of the distribution of *KRAS* mutations across all major nonHGSC subtypes. The frequency of *KRAS* mutations raised in the trend HGSC (1.2%) << clear cell (13%) < endometrioid (27%) = LGSC (28%) < mucinous (67%). Even more interesting was the trend in the ratio of *TP53*/*KRAS* mutability among subtypes, where mucinous and clear cell cases had a 1/1 ratio, while endometrioid and LGSC subtypes had more *KRAS* than *TP53* mutations. Patients with the HGSC subtype had a ratio close to 100/1 in favor of *TP53*. Most interestingly, analysis of the GENIE dataset revealed a considerable fraction of *TP53-KRAS* co-mutated patients, again with a high heterogeneity across subtypes. Almost half (46%) of patients with the mucinous subtype had both genes mutated. The other subtypes had a much lower proportion of such events, 4% for endometrioid and 1.5% for clear cell EOC. The occurrence of this phenomenon in LGSC and HGSC was comparable and less than 1% in both cases (**Fig. 3A**).

As GENIE does not contain expression data, we used the TCGA-OV dataset (n=374) for the assessment of transcript levels. The comparison of TP53 transcript levels with main mutation classification groups confirmed the trend observed in our study, i.e., significantly higher level in tumors harboring missense mutations (p=0.002) and lower in those with nonsense, frameshift, or splicing mutations of (p=0.009) compared to wild-type (**Fig. 3B**). For *KRAS*, no significant association of transcript expression with mutation spectra was found (p>0.05, **Fig. 3C**), perhaps due to the low number of observations (n=3 mutated samples). A weak, non-significant correlation was observed between TP53 and KRAS transcripts (p=0.052, **Fig. 3D**). In terms of available clinical data, neither grade (G1 or G2 versus G3 or G4) nor stage (stage I or II versus III or IV) were significantly associated with KRAS or TP53 transcript level (p>0.05, data not shown).

Only two *KRAS*-*TP53* co-mutations were found in the TCGA dataset. One patient (TCGA-29-1696-01A) had *KRAS* Gly12Arg with *TP53* frameshift co-mutation, stage IIIC, G2, and died 34 months after diagnosis. The second patient (TCGA-61-2009-01A) had *KRAS* Glu61Leu with *TP53* missense co-mutation, stage IIIC, G3, and was alive 40 months after diagnosis. Thus, external data do not seem to corroborate our observation of the considerably poorer prognosis of the three EOC patients with such co-mutations.

Due to the absence of survival data in the public version of GENIE and the lack of histopathologically confirmed subtype stratification in the TCGA dataset (however, all tumors were serous), we could not attempt the validation of our prognostic associations.

**Discussion**

The present study confirmed by a gold standard direct sequencing method the presence of mutations in clinically actionable EOC oncodrivers *TP53* and *KRAS*, reported by previous whole exome sequencing of 50 patients. We further screened both genes in the additional sample set of 127 EOC patients and complemented the somatic genotype with transcript expression data with the intent to provide deeper functional insight.

In general, results show that the functional classification of mutations and disease subtype context matters much more than carrier status alone. These aspects need careful investigation before any sensible clinical exploitation. Specifically, HGSC differs from other EOC subtypes by dominance of *TP53* mutations, while *KRAS* mutations are more relevant to nonHGSC subtypes. This observation agrees with the generally accepted view [20] and may have clinical consequences [21]. The prevalence of *KRAS* mutations in patients with less advanced stages I/II found by us complies with the fact that some nonHGSC subtypes are more frequently diagnosed with less advanced disease than HGSC ones [22]. Nevertheless, this was reflected by non-significantly better PFI only for clear cell subtype and just mild effect on general prognosis in terms of OS. However, the clear cell subtype is considered less sensitive to platinum-based chemotherapy than other EOCs [23] suggesting that our observation may be due to the low number of samples evaluated (n=10). The striking prevalence of *KRAS* mutations in nonHGSC subtypes calls for their integration into clinical trials with future *KRAS* inhibitors. Studies with *KRAS*-G12D (MRTX1133) [24] or pan-KRAS [25] inhibitors show promising results and the *KRAS*-G12C mutation inhibitors already have been approved for the personalization of therapy. Sotorasib has been approved for targeted therapy of non-small cell lung cancer (NSCLC) [26] and adagrasib for NSCLC and colorectal carcinoma [27].

The relevance of so-called loss-of-function or gain-of-function *TP53* variants [28] for disease progression and potential therapy targeting was discussed before [29]. Therefore, we used The TP53 database of and The Clinical Knowledgebase for the stratification of patients into subgroups with variants classified according to different functional effects (**Table 2**). Except for variants disrupting the DNA binding loop, being predictive for prolonged PFI, other stratifications had no clinical consequences.

Perhaps the most important result for contemporary considerations on targeted therapy and immunotherapy appeared after the analysis of *KRAS*-*TP53* co-mutated tumors. Previous studies in NSCLC [30,31] or pancreatic carcinoma [32] reported contradicting results. Despite both author groups demonstrating the dismal prognosis of patients harboring such alterations, the immunologically “hot” status has been claimed for NSCLC, while the “cold” status for pancreatic carcinoma with presumed consequences for the results of eventual immune checkpoint blockade therapy. Our study shows that EOC may be subject to further research in this area as co-mutated patients in the present study (n=4) had significantly worsened OS, moving EOC closer in this to pancreatic cancer.

The analysis of intratumoral TP53 and KRAS transcript levels has shown several significant results. First, they were mutually significantly correlated. Second, TP53 transcript level well mirrored its mutation status, namely tumors of missense variants carriers had higher, while those with other mutation types, classified as having a high predicted functional effect, lower levels compared to the wild-type (p<0.001). Accordingly, variants classified as loss-of-function had lower level than those with the gain-of-function status. Finally, yet importantly, nonHGSC tumors had significantly lower KRAS transcript levels than HGSC ones, while no correlation between mutation status and transcript level was found in this subgroup suggesting involvement of a more complex mechanism, e.g., epigenetics involvement. Both KRAS and p53 protein levels correlated with their respective transcript levels, further substantiating the functional relevance of transcript analysis.

External validation using the GENIE dataset (n=2210) [33] helped to validate the observed correlation between *TP53* mutation types and transcript expression. More importantly, it enabled a more precise evaluation of the distribution of *TP53* and *KRAS* mutation status across EOC subtypes. This analysis revealed an increasing trend in the ratio *TP53*/*KRAS* mutability: HGSC >> clear cell ≈ mucinous > endometrioid >> LGSC. An even more striking disproportion in *TP53*-*KRAS* co-mutation frequency: mucinous (53/115) >> endometrioid (9/223) > clear cell (4/265) > HGSC (14/1467) > LGSC (1/140) was apparent. Despite the common occurrence of co-mutations in the mucinous subtype was already described [34], both trends suggest enormous variability among EOC subtypes, which calls for exploitation in individualized therapy.

Several limitations of the present study need to be mentioned. Firstly, the sample size precludes robust analysis of nonHGSC subtypes, which are very rare (<10% of EOC each). Unfortunately, due to serious constraints in clinical data availability in both GENIE (missing survival) [33] and TCGA (missing EOC subtype), we could not externally validate our potentially clinically relevant results. Thus, more studies are necessary for this area and our study may contribute to meta-analyses. Second, patients analyzed in this study were untreated with PARPi or other targeted drugs. Platinum-based chemotherapy is considered the standard of care in EOC [35], and chemosensitivity to platinum and PARPi overlaps [36,37]. Thus, our sample set is still relevant from this point of view. Lastly, transcript levels may not robustly correlate with protein levels as assessed by immunohistochemistry. However, while immunohistochemistry is routinely clinically used for *TP53* assessment, it is not in the case of *KRAS*, where just the mutation status is considered for EGFR blockade therapy, and functional approval, especially for rarely occurring variants, is missing. Our study shows that this area needs further attention. Finally, the present study has clear benefits in ethnical homogeneity of the patient population, unified therapy regimen, and long-term complete clinical follow-up.

In conclusion, our study confirms previous data on *KRAS* as a valid and hopefully soon druggable target for nonHGSC EOCs and identifies the prognostic value of *TP53* mutations in the DNA binding loop for a fraction of patients. Furthermore, we describe an intriguing enrichment of *TP53-KRAS* co-mutations in the mucinous subtype of EOC based on analysis of an external dataset of 2210 samples. Our results further extend the area of precision oncology of EOC and suggest directions for future functional and preclinical studies.

**Patients and Methods**

*Patients*

For this study, we used samples of surgically resected, primary EOC tumors from 50 patients (confirmation set) with available whole exome data [14] and additional 127 EOC patients (validation set) without exome data. Patients were prospectively recruited at University Hospitals Motol, Královské Vinohrady (both in Prague, Czech Republic), and Pilsen (Czech Republic) between 2009 and 2020. Tumor samples were collected fresh and promptly frozen and stored at -80°C until isolation of nucleic acids. Peripheral blood samples were taken from all patients to enable tumor-normal matched analysis.

Collaborating clinicians collected the following clinical data on each patient: age at diagnosis, FIGO stage (pTNM), the histological subtype and grade of the tumor, presence of distant metastasis or residuum after surgery, oncological treatments, chemosensitivity status, and overall survival (OS) from medical records. The chemosensitivity status was based on the platinum-free interval (PFI) measured as the time from the end of the platinum-based adjuvant chemotherapy to disease recurrence or progression [38]. Patients having PFI≤6 months were considered platinum-resistant and patients with PFI≥12 months platinum-sensitive. Several patients had PFI in the range of 7-12 months and were classified as partially platinum-sensitive. These patients were tentatively included in the resistant group and all association analyses were performed both with and without them. Consensual results are provided. The OS was defined as the time elapsed between surgical resection and death of any cause or patient censoring. Detailed clinical characteristics of the patients are in **Table 1**.

Experimental protocol of the study was approved by the Institutional Review Boards of the National Institute of Public Health in Prague (approval reference no. IGA NS9803-4 of 2 February 2008), University Hospital Motol (approval reference no. EK-890/15 of 24 June 2015), University Hospital Královské Vinohrady (approval reference no. EK-VP/40/0/2017 of 28 June 2017), and University Hospital Pilsen (approval reference no. 16-29013A of 4 June 2015). All patients included in the study read and signed the Informed Consent of the Patient.

*Isolation of nucleic acids and cDNA synthesis*

DNA from peripheral blood lymphocytes was isolated using the DNeasy Blood and Tissue Kit (Qiagen, Hilden, Germany). Processing tumor tissue samples involved grinding them into a fine powder using a mortar and pestle under liquid nitrogen. Subsequently, we utilized the AllPrep DNA/RNA/Protein Mini Kit (Qiagen) according to the manufacturer's protocol for the isolation of total RNA and DNA. The quantity of the RNA and DNA samples was assessed using the Qubit 4 Nucleic Acid Fluorometric Quantification System (ThermoFisher Scientific, Waltham, MA, USA) and quality was checked by measuring the integrity number (RIN and DIN) using Agilent TapeStation 2200 (ThermoFisher Scientific). RNA was transcribed into cDNA with the help of the RevertAid™ First Strand cDNA Synthesis kit (ThermoFisher Scientific) according to the manufacturer's protocol and checked using the previously published method [39].

*Gene expression analysis*

Quantitative real-time PCR (qPCR) was performed using TaqMan® Gene Expression Assays (ThermoFisher), namely TP53 (Hs01034249_m1) and KRAS (Hs00364284_g1). PPIA (Hs99999904_m1), UBC (Hs00824723_m1), and YWHAZ (Hs03044281_g1), selected previously using NormFinder and geNorm software, served as reference genes for results normalization [40]. The reaction mixture with volume of 5 µL contained 1 µL of 5× Hot FirePol Probe qPCR Mix Plus (ROX) (Solis BioDyne OÜ, Tartu, Estonia), 0.25 µL of 20× TaqMan® Gene Expression Assay specified above, 1.75 µL of nuclease-free water, and 2 µL of 8-times diluted cDNA. qPCR reactions were performed in a 384-well block of the ViiA7 Real-Time PCR System and evaluated using the ViiA7 System Software (Life Technologies, Carlsbad, CA, USA). Cycling parameters were initially held at 50 ◦C for 2 min and 10 min denaturation at 95 ◦C, followed by 45 cycles consisting of 15 sec of denaturation at 95 ◦C and 60 sec of annealing/extension at 60 ◦C. The non-template control contained water instead of cDNA and negative cDNA synthesis controls (RNA transcribed without reverse transcriptase) were employed to control carry-over contamination. All samples were analyzed in duplicates and samples with a standard deviation > 0.5 Ct between replicates were re-analyzed. The qPCR process adhered to the Minimum Information for Publication of Quantitative Real-Time PCR Experiments Guidelines (MIQE) [41].

Differences between samples and groups of patients were calculated from raw Ct values with the comparative Ct method described previously [42]. The 2^−∆Ct^ method was used for relative quantification of gene expression, and the 2^−∆∆Ct^ method was used for fold change calculation in groups divided by clinical data or mutation carriage or classification.

*Direct sequencing*

Exons 2 and 3 of *KRAS* and 4-10 of *TP53* were subjected to direct sequencing using the Sanger method. Briefly, DNA was amplified between oligonucleotide primer pairs specific for each amplicon (**Online Resource 7**) using regular PCR and after product length verification on agarose gel purified by ethanol precipitation. Each reaction was optimized to produce a strong single-band product. Sequencing reactions were then performed using the BigDye Terminator v3.1 Cycle Sequencing Kit (Invitrogen) with approximately 10 ng of PCR product and 2 pmol of sequencing primer in 10 µl final reaction volume according to the producer’s protocol. Separate sequencing reactions were run with both forward and reverse sequencing primers (**Online Resource 7**). The acquired products were purified using ExoSAP-IT™ PCR Product Cleanup Reagent (Applied Biosystems, Foster City, CA). DNA sequencing was performed by a capillary electrophoresis-based system commercially (SEQme, s.r.o., Dobris, Czech Republic). Raw results were evaluated by BioEdit 7.2.5 program and Sequencing Analysis Software v5.2 (Applied Biosystems). Oligonucleotide primers (**Online Resource 7**) for sequencing were designed using the Primer3 software [43].

*Immunoblotting*

52 tumor samples representing highest and lowest transcript levels and wide spectra of mutations in *KRAS* and *TP53* were selected for protein study. Proteins isolated from patient tissues using the AllPrep DNA/RNA/Protein Mini Kit (Qiagen) were quantified using the Pierce™ BCA Protein Assay Kit (Thermo Fisher Scientific). In addition to patient tissue samples, we added a protein sample from the Panc-1 cell line to each of the four membranes. Panc-1 has been previously tested for high expression of KRAS and p53 and served as a standard for inter-membrane normalization of detected expression levels. A total of 20 μg of protein was loaded onto a 12 % (w/v) polyacrylamide gel, separated by SDS-PAGE, and subsequently transferred onto a nitrocellulose membrane. Following transfer, the membrane was cut into two sections. The upper section was blocked for 60 min in Blocker™ BLOTTO in TBS buffer (Thermo Fisher Scientific) and then incubated overnight at 4 °C with a multiplex combination of primary antibodies against p53 (mouse monoclonal antibody, A10610, Abclonal Science, Düsseldorf, Germany) and GAPDH (used as a loading control; rabbit monoclonal antibody, 14C10, #2118, Cell Signaling Technology, MA, USA), both diluted in Blocker™ BLOTTO in TBS containing 0.2% (v/v) Tween®20. The lower section was incubated with a primary antibody against KRAS (rabbit polyclonal antibody, GTX100636, GeneTex, CA, USA) under the same conditions. After rinsing with TBS containing 0.1 % (v/v) Tween®20, the membrane sections were incubated for 60 min at room temperature with secondary antibodies. The upper section was probed with IRDye® 800CW Goat anti-Mouse IgG and IRDye® 680RD Goat anti-Rabbit IgG (Li-Cor, Lincoln, NE, USA), while the lower section was incubated with IRDye® 800CW Goat anti-Rabbit IgG. Proteins were visualized using the Odyssey® Fc Imaging System (Li-Cor) and quantified using Image Studio version 4.0.21 (Li-Cor). Membranes with areas extracted for Fig. 1D preparation are in **Online Resource 8.**

*External datasets*

For validation of somatic variants in *TP53* and *KRAS*, the American Association for Cancer Research (AACR) Genomics Evidence Neoplasia Information Exchange (GENIE) 15.0-public release dataset (released on 1 Feb, 2024), composed of tumor panel sequencing data from multiple major cancer centers, was utilized [33]. Only samples fulfilling the following criteria were used: EOC, primary tumor, any somatic mutation data found after matching by sample ID, and gene of interest (*TP53* and/or *KRAS*) included in the respective sequencing panel (final dataset: n=2210). For validation of expression levels and mutation data, we used the RNAseq gene expression (FPKM-UQ normalized) and DNAseq mutation data of the GDC TCGA-OV cohort (Mutect2 pipeline), downloaded from the University of California Santa Cruz Xenabrowser portal (https://xenabrowser.net), which were then filtered to only primary ovarian tumors (n=374). The dataset does not contain minority subtypes, nor detailed histopathological annotation, with all samples being classified merely as serous, and therefore, subtype-sensitive analyses were not performed using this dataset.

*Statistical analysis*

Associations of categorical clinical data of patients (stage, grade of tumor, residuum, chemosensitivity status) with functional classification of mutations were analyzed using the Pearson chi-square or Fisher’s exact test. For the evaluation of associations of continuous variables such as age at diagnosis or transcript expression with categorical ones, the Kruskal-Wallis test was used. Correlations among continuous variables were tested with Spearman’s rho correlation. All tests were two-sided and p-values <0.05 were considered statistically significant. Survival curves were plotted using the Kaplan-Meier method. Expression levels were distributed by quartiles and the “optimal cut-off” was defined as the highest statistical significance by the log-rank test. All statistical analyses were performed using the SPSS v16 program (SPSS, Chicago, IL, USA). Previous version of the manuscript was posted to the ResearchSquare [44] and the current paper presents thoroughly revised content.

**List of Abbreviations**

AACR – The American Association for Cancer Research

CKB – The Clinical Knowledgebase

EOC – epithelial ovarian cancer

FIGO – The International Federation of Gynecology and Obstetrics

FOLFOX – 5-fluorouracil, leucovorin, and oxaliplatin

GENIE – Genomics Evidence Neoplasia Information Exchange

HGSC – high-grade serous ovarian carcinomas

LGSC – low-grade serous ovarian carcinomas

MIQE – the Minimum Information for Publication of Quantitative Real-Time PCR Experiments Guidelines

NGS – next generation sequencing

nonHGSC – other than high-grade serous carcinoma

NSCLC – non-small cell lung cancer

OS – overall survival

PARPi – poly(ADP-ribose) polymerase inhibitors

PFI – platinum-free interval

qPCR – quantitative real-time polymerase chain reaction

**Statements and Declarations**

**Ethics approval and consent to participate**

Experimental protocol of the study was approved by the Institutional Review Boards of the National Institute of Public Health in Prague (approval reference no. IGA NS9803-4 of 2 February 2008), University Hospital Motol (approval reference no. EK-890/15 of 24 June 2015), University Hospital Kralovske Vinohrady (approval reference no. EK-VP/40/0/2017 of 28 June 2017), and University Hospital Pilsen (approval reference no. 16-29013A of 4 June 2015). All patients included in the study read and signed the Informed Consent of the Patient.

**Consent for publication**

Not applicable.

**Data Availability Statement**

The authors confirm that molecular data supporting the findings of this study are available within the article and its supplementary materials. Individual clinical data are not publicly available due to restrictions on information that could compromise the privacy of research participants.

**Competing Interests**

The authors declare no conflict of interest. The funders had no role in the design of the study; in the collection, analyses, or interpretation of data; in the writing of the manuscript, or in the decision to publish the results.

**Funding**

This work was funded by the Czech Health Research Council grant no. NU22-08-00186 to P.S. and the Grant Agency of Charles University, project no. 307123 to M.O.A.

**Author Contributions**

Conceptualization – R.V. & P.S.

Methodology – M.A., E.A., I.K., V.H., A.S., & F.A.

Formal Analysis – P.H., R.V., & P.S.

Investigation – M.A., E.A., V.H., A.S., & I.K.

Resources – L.R., M.H., M.M., K.K., A.B., & J.B.

Writing – Original Draft – M.A., R.V., & P.S.

Writing – Review & Editing – M.A., E.A., I.K., P.H., V.H., F.A., L.R., M.H., M.M., K.K., A.B., J.B., A.S., R.V. & P.S.

Visualization – P.H., A.S., & P.S.

Supervision – R.V. & P.S.

Project Administration – P.S.

Funding Acquisition – P.S.

**Acknowledgements**

The authors would like to thank all participating patients for their kind consent to the study and the clinical personnel for outstanding support.

**5. References**

1. Cabasag CJ, Fagan PJ, Ferlay J, Vignat J, Laversanne M, Liu L, et al. Ovarian cancer today and tomorrow: A global assessment by world region and Human Development Index using GLOBOCAN 2020. *Int J Cancer*. 2022;151(9):1535-1541. doi: 10.1002/ijc.34002.

2. Matulonis UA, Sood AK, Fallowfield L, Howitt BE, Sehouli J, Karlan BY. Ovarian cancer. *Nat Rev Dis Primers* 2016;2:16061. doi: 10.1038/nrdp.2016.61.

3. Matz M, Coleman MP, Carreira H, Salmerón D, Chirlaque MD, Allemani C; CONCORD Working Group. Worldwide comparison of ovarian cancer survival: Histological group and stage at diagnosis (CONCORD-2). *Gynecol Oncol*. 2017;144(2):396-404. doi:10.1016/j.ygyno.2016.11.019.

4. Kim A, Ueda Y, Naka T, Enomoto T. Therapeutic strategies in epithelial ovarian cancer. *J Exp Clin Cancer Res.* 2012;31(1):14. doi:10.1186/1756-9966-31-14.

5. Ovarian Cancer Survival Rates | Ovarian Cancer Prognosis. Accessed February 15, 2024. https://www.cancer.org/cancer/types/ovarian-cancer/detection-diagnosis-staging/survival-rates.html.

6. Lheureux S, Gourley C, Vergote I, Oza AM. Epithelial ovarian cancer. *Lancet*. 2019;393(10177):P1240-1253. doi: 10.1016/S0140-6736(18)32552-2.

7. Lisio MA, Fu L, Goyeneche A, Gao ZH, Telleria C. High-Grade Serous Ovarian Cancer: Basic Sciences, Clinical and Therapeutic Standpoints. *Int J Mol Sci*. 2019;20(4):952. doi:10.3390/ijms20040952.

8. Cortez AJ, Tudrej P, Kujawa KA, Lisowska KM. Advances in ovarian cancer therapy. *Cancer Chemother Pharmacol*. 2018;81(1):17-38. doi:10.1007/s00280-017-3501-8.

9. O’Sullivan Coyne G, Chen AP, Meehan R, Doroshow JH. PARP Inhibitors in Reproductive System Cancers: Current Use and Developments. *Drugs*. 2017;77(2):113-130. doi:10.1007/s40265-016-0688-7.

10. Banerjee S, Gonzalez-Martin A, Harter P, Lorusso D, Moore KN, Oaknin A, Ray-Coquard I. First-line PARP inhibitors in ovarian cancer: summary of an ESMO Open - Cancer Horizons round-table discussion. *ESMO Open*. 2020;5(6):e001110. doi:10.1136/esmoopen-2020-001110.

11. Chartron E, Theillet C, Guiu S, Jacot W. Targeting homologous repair deficiency in breast and ovarian cancers: Biological pathways, preclinical and clinical data. *Crit Rev. Oncol Hematol*. 2019;133:58-73. doi:10.1016/j.critrevonc.2018.10.012.

12. Rojas V, Hirshfield KM, Ganesan S, Rodriguez-Rodriguez L. Molecular Characterization of Epithelial Ovarian Cancer: Implications for Diagnosis and Treatment. *Int J Mol Sci*. 2016;17(12):2113. doi:10.3390/ijms17122113.

13. Goyal G, Fan T, Silberstein PT. Hereditary cancer syndromes: utilizing DNA repair deficiency as therapeutic target. *Fam Cancer*. 2016;15(3):359-366. doi:10.1007/s10689-016-9883-7.

14. Hlaváč V, Holý P, Václavíková R, Rob L, Hruda M, Mrhalová M, et al. Whole-exome sequencing of epithelial ovarian carcinomas differing in resistance to platinum therapy. *Life Sci Alliance*. 2022;5(12):e202201551. doi:10.26508/lsa.202201551.

15. Norquist BM, Brady MF, Harrell MI, Walsh T, Lee MK, Gulsuner S, et al. Mutations in homologous recombination genes and outcomes in ovarian carcinoma patients in GOG 218: An NRG oncology/gynecologic oncology group study. *Clin Cancer Res* 2018;24:777–783. doi:10.1158/1078-0432.CCR-17-1327.

16. Zhang H, Liu T, Zhang Z, Payne SH, Zhang B, McDermott JE, et al. Integrated Proteogenomic Characterization of Human High-Grade Serous Ovarian Cancer. *Cell*. 2016;166(3):755-765. doi:10.1016/j.cell.2016.05.069

17. Li C, Bonazzoli E, Bellone S, Choi J, Dong W, Menderes G, et al. Mutational landscape of primary, metastatic, and recurrent ovarian cancer reveals c-MYC gains as potential target for BET inhibitors. *Proc Natl Acad Sci U S A* 2019;116:619–624. doi: 10.1073/pnas.1814027116.

18. de Witte CJ, Kutzera J, van Hoeck A, Nguyen L, Boere IA, Jalving M, et al. Distinct Genomic Profiles Are Associated with Treatment Response and Survival in Ovarian Cancer. *Cancers (Basel)* 2022;14:1511. doi: 10.3390/cancers14061511.

19. Pejovic T, Fitch K, Mills G. Ovarian cancer recurrence: "is the definition of platinum resistance modified by PARP inhibitors and other intervening treatments?". *Cancer Drug Resist*. 2022;5:451-458. doi: 10.20517/cdr.2021.138.

20. Nasioudis D, Fernandez ML, Wong N, Powell DJ Jr, Mills GB, Westin S, et al. The spectrum of MAPK-ERK pathway genomic alterations in gynecologic malignancies: Opportunities for novel therapeutic approaches. *Gynecol Oncol*. 2023;177:86-94. doi: 10.1016/j.ygyno.2023.08.007.

21. Therachiyil L, Anand A, Azmi A, Bhat A, Korashy HM, Uddin S. Role of RAS signaling in ovarian cancer. *F1000Res*. 2022;11:1253. doi: 10.12688/f1000research.126337.1.

22. Peres LC, Cushing-Haugen KL, Köbel M, Harris HR, Berchuck A, et al. Invasive Epithelial Ovarian Cancer Survival by Histotype and Disease Stage. *J Natl Cancer Inst.* 2019;111(1):60-68. doi: 10.1093/jnci/djy071.

23. Stewart J, Cunningham N, Banerjee S. New therapies for clear cell ovarian carcinoma. *Int J Gynecol Cancer*. 2023;33(3):385–93. doi:10.1136/ijgc-2022-003704.

24. Tang D, Kang R. Glimmers of hope for targeting oncogenic KRAS-G12D. *Cancer Gene Ther* 2023; 30, 391–393. doi:10.1038/s41417-022-00561-3.

25. Kim D, Herdeis L, Rudolph D, Zhao Y, Böttcher J, Vides A, et al. Pan-KRAS inhibitor disables oncogenic signalling and tumour growth. *Nature* 2023; 619, 160–166. doi:10.1038/s41586-023-06123-3.

26. Nakajima EC, Drezner N, Li X, Mishra-Kalyani PS, Liu Y, Zhao H, et al. FDA Approval Summary: Sotorasib for KRAS G12C-Mutated Metastatic NSCLC. *Clin Cancer Res.* 2022;28(8):1482-1486. doi: 10.1158/1078-0432.CCR-21-3074.

27. Dhillon S. Adagrasib: First Approval. *Drugs*. 2023;83(3):275-285. doi: 10.1007/s40265-023-01839-y.

28. Brachova P, Mueting SR, Carlson MJ, Goodheart MJ, Button AM, Mott SL, et al. TP53 oncomorphic mutations predict resistance to platinum‑ and taxane‑based standard chemotherapy in patients diagnosed with advanced serous ovarian carcinoma. *Int J Oncol*. 2015;46(2):607-18. doi: 10.3892/ijo.2014.2747.

29. Peuget S, Zhou X, Selivanova G. Translating p53-based therapies for cancer into the clinic. *Nat Rev Cancer*. 2024;24(3):192-215. doi: 10.1038/s41568-023-00658-3.

30. Dong ZY, Zhong WZ, Zhang XC, Su J, Xie Z, Liu SY, et al. Potential Predictive Value of *TP53* and *KRAS* Mutation Status for Response to PD-1 Blockade Immunotherapy in Lung Adenocarcinoma. *Clin Cancer Res*. 2017;23(12):3012-3024. doi: 10.1158/1078-0432.CCR-16-2554.

31. Gu M, Xu T, Chang P. *KRAS/LKB1* and *KRAS/TP53* co-mutations create divergent immune signatures in lung adenocarcinomas. *Ther Adv Med Oncol*. 2021;13:17588359211006950. doi: 10.1177/17588359211006950.

32. Datta J, Bianchi A, De Castro Silva I, Deshpande NU, Cao LL, Mehra S, et al. Distinct mechanisms of innate and adaptive immune regulation underlie poor oncologic outcomes associated with KRAS-TP53 co-alteration in pancreatic cancer. *Oncogene*. 2022;41(28):3640-3654. doi: 10.1038/s41388-022-02368-w.

33. AACR Project GENIE Consortium. AACR Project GENIE: Powering Precision Medicine through an International Consortium. *Cancer Discov*. 2017;7(8):818-831. doi: 10.1158/2159-8290.CD-17-0151

34. Rechsteiner M, Zimmermann AK, Wild PJ, Caduff R, von Teichman A, et al. TP53 mutations are common in all subtypes of epithelial ovarian cancer and occur concomitantly with KRAS mutations in the mucinous type. *Exp Mol Pathol*. 2013;95(2):235-41. doi: 10.1016/j.yexmp.

35. Soberanis Pina P, Lheureux S. Overcoming PARP inhibitor resistance in ovarian cancer. *Int J Gynecol Cancer.* 2023;33(3):364-376. doi: 10.1136/ijgc-2022-003698.

36. Coelho R, Tozzi A, Disler M, Lombardo F, Fedier A, López MN, et al. Overlapping gene dependencies for PARP inhibitors and carboplatin response identified by functional CRISPR-Cas9 screening in ovarian cancer. *Cell Death Dis*. 2022;13(10):909. doi: 10.1038/s41419-022-05347-x.

37. McMullen M, Karakasis K, Madariaga A, Oza AM. Overcoming Platinum and PARP-Inhibitor Resistance in Ovarian Cancer. *Cancers* 2020;12:1607. doi:10.3390/cancers12061607.

38. Friedlander M, Trimble E, Tinker A, Alberts D, Avall-Lundqvist E, Brady M, et al. Clinical trials in recurrent ovarian cancer. *Int J Gynecol* *Cancer* 2011;21:771–775. doi:10.1097/IGC.0b013e31821bb8aa.

39. Soucek P, Anzenbacher P, Skoumalova I, Dvorak M. Expression of cytochrome P450 genes in CD34+ hematopoietic stem and progenitor cells. *Stem Cells* 2005; 23:1417‑1422. doi: 10.1634/stemcells.2005-0066.

40. Elsnerova K, Mohelnikova-Duchonova B, Cerovska E, Ehrlichova M, Gut I, Rob L, et al. Gene expression of membrane transporters: Importance for prognosis and progression of ovarian carcinoma. *Oncol Rep.* 2016;35:2159–70. doi: 10.3892/or.2016.4599.

41. Bustin SA, Benes V, Garson JA, Hellemans J, Huggett J, Kubista M, et al. The MIQE guidelines: minimum information for publication of quantitative real-time PCR experiments. *Clin Chem* 2009;55:611–22. doi: 10.1373/clinchem.2008.112797.

42. Livak KJ, Schmittgen TD. Analysis of relative gene expression data using real-time quantitative PCR and the 2(-Delta Delta C(T)) Method. *Methods* 2001;25:402–408. doi: 10.1006/meth.2001.1262.

43. Untergasser A, Cutcutache I, Koressaar T, Ye J, Faircloth BC, Remm M, et al. Primer3—new capabilities and interfaces. *Nucleic Acids Res.* 2012;40:e115–e115. doi: 10.1093/nar/gks596.

44. Mohammad Al Obeed Allah, Esraa Ali, Ivona Krus et al. Functional validation of somatic variability in TP53 and KRAS for prediction of platinum sensitivity and prognosis in epithelial ovarian carcinoma patients, 22 October 2024, PREPRINT (Version 1) available at Research Square [https://doi.org/10.21203/rs.3.rs-5224537/v1].

**Figure legends**

**Fig. 1** Associations of TP53 and KRAS normalized transcript levels in tumors with characteristics of EOC patients

(A) TP53 normalized transcript level with *TP53* mutation type or (B) effect (gain-of-function vs. loss-of-function). (C) Mutual correlation between KRAS and TP53 transcript levels. (D) Four membranes representing KRAS and p53 protein levels with GAPDH loading control and Panc-1 cell line for inter-membrane normalization. (E) KRAS normalized transcript level with EOC subtype and (F) mutation status.

High impact means nonsense, frameshift, or splice site functional variant classification.

Footnote: *5 high impact and 4 missense variants could not be classified and are excluded from the plot.

**Fig. 2** Associations between patient survival and carriage of *TP53* or *KRAS* mutations

(A) Platinum-free interval stratified by carriage of *TP53* DNA binding loop mutations in EOC patients and (B) overall survival and platinum-free interval (C) in *TP53*-*KRAS* co-mutated patients compared to wild-type or single gene mutated EOC patients.

**Fig. 3** Validation of *TP53* and *KRAS* mutational spectra and intratumoral transcript levels in EOC subtypes using external datasets

(A) *TP53* and *KRAS* mutational spectra in EOC subtypes. (B) TP53 and (C) KRAS intratumoral transcript levels stratified their by mutation type or classification. (D) Correlation between TP53 and KRAS intratumoral transcript levels (lack of mutual correlation).

**Table 1:** Clinical characteristics of EOC patients

| **Parameters** | **Number of patients** | **Percentage** |
| --- | --- | --- |
| **Age at diagnosis** | 177 | 100 |
| Median ± SD (years) | 62.0 ± 11.5 |  |
| **FIGO stage** |  |  |
| I | 12 | 7 |
| II | 10 | 6 |
| III | 139 | 82 |
| IV | 8 | 5 |
| Data not available | 8 | ̶̶ |
| **Histologic grade (G)** |  |  |
| G1 | 11 | 6 |
| G2 | 15 | 9 |
| G3 | 146 | 85 |
| Gx | 5 | ̶̶ |
| **Tumor subtype** |  |  |
| HGSC | 143 | 84 |
| Other* | 28 | 16 |
| Data not available | 6 | ̶̶ |
| **Distant metastasis** |  |  |
| Absent | 161 | 95 |
| Present | 8 | 5 |
| Data not available | 8 | ̶̶ |
| **Neoadjuvant chemotherapy** |  |  |
| Administered | 57 | 32 |
| Not administered | 120 | 68 |
| **Residuum after surgery** |  |  |
| Present** | 85 | 50 |
| Absent (R0) | 85 | 50 |
| Data not available | 7 | ̶̶ |
| **Adjuvant chemotherapy** |  |  |
| Platinum-based^#^ | 165 | 96 |
| Taxane monotherapy | 2 | 1 |
| Not administered | 4 | 3 |
| Data not available | 6 | ̶̶ |
| **Chemosensitivity status** |  |  |
| Resistant (PFI ≤ 6 months) | 38 | 23 |
| Intermediate (PFI 7-11 months) | 24 | 15 |
| Sensitive (PFI ≥ 12 months) | 101 | 62 |
| Data not available | 14 | ̶̶ |
| **Platinum-free interval** | 168 | 95 |
| Median ± 95% confidence interval (months) | 25 | 17.9-32.1 |
| **Overall survival** | 168 | 95 |
| Median ± 95% confidence interval (months) | 48 | 37.7 - 58.3 |

Footnotes:

*Other subtypes include the following carcinomas: mucinous (n=9), clear cell (n=10), low grade serous (n=5), endometrioid (n=2), and borderline (n=2).

**Includes all ratings above R0 (R1, R2, unspecified).

^#^Platinum-based chemotherapy regimens include n=147 taxane (paclitaxel/docetaxel) with platinum (carboplatin/cisplatin), n=9 platinum monotherapy, other (n=1 FOLFOX, n=1 platinum with anthracycline, n=1 platinum with paclitaxel and anthracycline, and n=6 platinum with paclitaxel and cyclophosphamide). FOLFOX = 5-fluorouracil, leucovorin, and oxaliplatin.

**Table 2:** Molecular characteristics of EOC patients

| **Gene** | **Number of patients** | **Percentage** |
| --- | --- | --- |
| ***KRAS* mutation status*** |  |  |
| *KRAS* wild-type | 161 | 93 |
| *KRAS* mutated | 15 | 8 |
| ***KRAS* mutation spectrum** |  |  |
| p.Gly12Asp | 5 | 33 |
| p.Gly12Val | 5 | 33 |
| p.Gln61His | 2 | 13 |
| p.Gly12Cys | 1 | 7 |
| p.Gly12Ala | 1 | 7 |
| p.Gln61Arg | 1 | 7 |
| ***TP53* mutation status** |  |  |
| *TP53* wild-type | 72 | 41 |
| *TP53* mutated | 105 | 59 |
| ***TP53* mutation spectrum** |  |  |
| *Hotspots* |  |  |
| p.Arg175His | 8 |  |
| p.Tyr220Cys | 7 |  |
| p.Arg273His | 6 |  |
| p.Arg248Gln | 5 |  |
| p.Arg248Trp | 4 |  |
| p.Arg282Trp | 4 |  |
| p.His214Arg | 3 |  |
| p.His179Gln | 2 |  |
| p.Arg249Trp | 2 |  |
| p.Cys275Phe | 2 |  |
| p.Gly279Glu | 2 |  |
| p.Glu198Ter | 3 |  |
| p.Arg213Ter | 3 |  |
| *Private missense mutations* | 32 |  |
| *Private frameshift or nonsense mutations* | 17 |  |
| *Private splice site mutations with pathogenic features* | 5 |  |
| ***TP53* mutation functional consequences**^#^ |  |  |
| Loss-of-function | 87 | 92 |
| Gain-of-function | 8 | 8 |
| Not classified | 10 | ̶̶ |
| *Dominant-negative effect (DNE) & loss-of-function (LOF) properties*^#^ | |  |
| DNE_LOF | 66 | 86 |
| notDNE_notLOF | 5 | 7 |
| notDNE_LOF | 5 | 7 |
| Not classified | 29 | ̶̶ |
| *Transactivation function*^#^ |  |  |
| non-functional | 64 | 90 |
| functional or partially functional | 7 | 10 |
| Not classified | 34 | ̶̶ |
| *DNA binding loop affected*^#^ |  |  |
| yes | 94 | 94 |
| no | 6 | 6 |
| Not applicable | 5 | - |

Footnotes:

*Result for two samples not available due to DNA of low quality/quantity.

^#^Evaluated using The TP53 database of NCI (https://tp53.isb-cgc.org/) and The Clinical Knowledgebase (https://ckb-core.genomenon.com/) and literature cited therein.

**Table 3:** Associations between *KRAS* mutational status and stage or tumor subtype of EOC patients

| **Characteristics** | ***KRAS* wild-type*** | ***KRAS* mutated*** | **p-value** |
| --- | --- | --- | --- |
| Stage I/II | 16 | 6 | 0.002 |
| Stage III/IV | 139 | 7 |  |
| HGSC | 139 | 4 | <0.001 |
| other subtypes | 18 | 10 |  |
|  | ***TP53* wild-type*** | ***TP53* mutated*** | **p-value** |
| HGSC | 50 | 93 | 0.002 |
| other subtypes | 19 | 9 |  |

Footnotes:

*Numbers of patients; for some patients clinical data or *KRAS* mutation status were not available.
